# Analyzing and predicting short-term substance use behaviors of persons who use drugs in the great plains of the U.S.

**DOI:** 10.1371/journal.pone.0312046

**Published:** 2024-11-27

**Authors:** Nguyen Thach, Patrick Habecker, Bergen Johnston, Lillianna Cervantes, Anika Eisenbraun, Alex Mason, Kimberly Tyler, Bilal Khan, Hau Chan

**Affiliations:** 1 School of Computing, University of Nebraska-Lincoln, Lincoln, Nebraska, United States of America; 2 Department of Sociology, University of Nebraska-Lincoln, Lincoln, Nebraska, United States of America; 3 Rural Drug Addiction Research Center, University of Nebraska-Lincoln, Lincoln, Nebraska, United States of America; 4 College of Education and Human Sciences, University of Nebraska-Lincoln, Lincoln, Nebraska, United States of America; 5 P.C. Rossin College of Engineering and Applied Science, Lehigh University, Bethlehem, Pennsylvania, United States of America; University of Toronto, CANADA

## Abstract

**Background:**

Substance use induces large economic and societal costs in the U.S. Understanding the change in substance use behaviors of persons who use drugs (PWUDs) over time, therefore, is important in order to inform healthcare providers, policymakers, and other stakeholders toward more efficient allocation of limited resources to at-risk PWUDs.

**Objective:**

This study examines the short-term (within a year) behavioral changes in substance use of PWUDs at the population and individual levels.

**Methods:**

237 PWUDs in the Great Plains of the U.S. were recruited by our team. The sample provides us longitudinal survey data regarding their individual attributes, including drug use behaviors, at two separate time periods spanning 4-12 months. At the population level, we analyze our data quantitatively for 18 illicit drugs; then, at the individual level, we build interpretable machine learning logistic regression and decision tree models for identifying relevant attributes to predict, for a given PWUD, (i) which drug(s) they would likely use and (ii) which drug(s) they would likely increase usage within the next 12 months. All predictive models were evaluated by computing the (averaged) Area under the Receiver Operating Characteristic curve (AUROC) and Area under the Precision-Recall curve (AUPR) on multiple distinct sets of hold-out sample.

**Results:**

At the population level, the extent of usage change and the number of drugs exhibiting usage changes follow power-law distributions. At the individual level, AUROC’s of the models for the top-4 prevalent drugs (marijuana, methamphetamines, amphetamines, and cocaine) range 0.756-0.829 (+2.88-7.66% improvement with respect to baseline models using only current usage of the respective drugs as input) for (i) and 0.670-0.765 (+4.34-18.0%) for (ii). The corresponding AUPR’s of the said models range 0.729-0.947 (+2.49-13.6%) for (i) and 0.348-0.618 (+26.9-87.6%) for (ii).

**Conclusion:**

The observed qualitative changes in short-term substance usage and the trained predictive models for (i) and (ii) can potentially inform human decision-making toward efficient allocation of appropriate resources to PWUDs at highest risk.

## Introduction

Substance use can create short-term and long-term negative consequences for persons who use drugs (PWUDs) [1–3]. These consequences include mental illness, HIV/AIDS, hepatitis, drug overdose, and death [1–3]. According to a 2021 National Survey on Drug Use and Health (NSDUH) from the Substance Abuse and Mental Health Services Administration (SAMHSA) [4], an estimated 161.8 million people aged 12 or older used a substance (out of which 54.7 million used tobacco, 133.1 million used alcohol, 31.6 million used marijuana, and 40.0 million used an illicit drug) in the past month before the NSDUH interview. Substance overdose deaths, including those related to methamphetamine, cocaine, heroin, and opioids, in the U.S. continue to increase, with over 106,000 deaths in 2021 [5]. Furthermore, substance use has induced a large economic cost in the U.S. including substance use-related crimes, healthcare, and loss of work productivity. According to the National Institute on Drug Abuse (NIDA), the economic cost of substance abuse in the U.S. exceeds $700 billion each year [6, 7]. In a detailed 2019 report [8] that accounts for economic loss and societal harm, the estimated cost is $3.73 trillion annually in the U.S. Of these, $98 billion, $119 billion, and $207 billion are due to crimes, healthcare, and productivity loss, respectively.

To help PWUDs and reduce the economic and societal costs of substance use, many organizations provide intervention and outreach programs/resources (e.g., rehabs, consulting services, and medical aids) for PWUDs, with the main goal of reducing and eliminating their usage of certain substances. While these programs and resources have shown to be effective to some extent [9, 10], they often require volunteer participation from PWUDs, who face the difficulties and reluctances of (self-)evaluating and (self-)determining whether they want or need help. Even when PWUDs agree to participate in these programs/resources, they may have already experienced prior harms such as overdose and mental illness. Therefore, it is important to prevent harm from occurring in the first place by carefully identifying PWUDs at the highest risk (As defined shortly, we consider “at risk” individuals as those who would escalate usage of one or more distinct drugs in the short term i.e., within a year.) and allocating them appropriate resources to reduce or eliminate potential harms.

PWUDs exhibit different substance use behaviors, including types, combinations, or number of drugs they use and the extent (e.g., once a month, once a week, etc.) they use different types of drugs. These behaviors are highly dynamic [11] and linked to many individual attributes (e.g., the onset of drug use and cessation history). As a result of the behavioral changes, PWUDs can become at risk in the future because of the potential increase in their usage of substances over time [11, 12]. While some PWUDs have the potential to decrease their usage and may not require extensive intervention, at-risk PWUDs that increase their substance usage might require additional attention and consideration as such behavior could lead to substance abuse, addictions, disorders, or other negative consequences in the future [11, 12]. Therefore, it is important to examine and understand the changes in substance use behaviors of PWUDs over time, especially those that have a pattern of increased substance use.

Yet, to date, such behavioral changes for any population group across any given short-term period (i.e., a few months to a year) remain unclear. There is a paucity of research on generative/descriptive models (for analyzing population substance use changes) or analytical tools (for predicting individual substance use changes) to help identify PWUDs that are at risk. Therefore, in this paper, we have two main objectives associated with understanding the change in substance use. At the population level, our first objective aims to investigate the extent of the qualitative changes in substance usage of PWUDs over time. At the individual level within the population, our second objective seeks to identify the key predictive attributes that can lead to individual changes in substance usage.

To achieve the objectives, our team collected surveys from a sample of 237 (anonymized) PWUDs in the Great Plains of the U.S. Using the data, for the first objective, we analyze the changes in substance use behaviors for 18 non-injection and injection drugs (e.g., marijuana, meth, amphetamines, cocaine, opioids, injection meth, etc.) at the population level. Following these observations, for the second objective, we examine the changes at the individual level by identifying (predictive) factors that can lead to or predict the (short-term) changes in substance use for PWUDs within the population. Toward identifying predictive factors, we use two types of interpretable machine learning models, logistic regression and decision tree, to study two predictive questions: within the next 12 months, (i) which drug(s) a PWUD would (likely) use and (ii) which drug(s) a PWUD would (likely) increase its usage. The resulting observations and trained models may be utilized by healthcare agencies, local communities, policymakers, and other stakeholders for better understanding of the changes in substance use of different drugs within a large population *and* for forming a decision aid while identifying the most vulnerable individuals and/or determining the most suitable resources to allocate to them.

## Materials and methods

### Background and related work

#### Data on substance use

A majority of substance use data in the U.S. is generated through cross-sectional studies that draw new samples each year for fielding a survey. SAMHSA-sponsored projects such as the National Survey of Drug Use and Health (NSDUH) and the Treatment Episode Data Set (TEDS); Centers for Disease Control and Prevention (CDC) projects such as the Behavioral Risk Factor Surveillance System (BRFSS) and the Youth Risk Behavior Surveillance System (YRBSS); and National Institutes of Health (NIH) projects like Monitoring the Future (MTF) are all primarily focused on cross-sectional samples and tracking incidence and change among the general population of the U.S. from year to year. Although rich in data, unlike the data collected by our team, these types of studies cannot follow an individual through time to understand how their experiences change, and the mechanisms associated with those changes. This type of work requires longitudinal and cohort studies that follow individuals through time and repeatedly ask the same people questions at different time points. The costs associated with maintaining contact with participants over time can be expensive, which makes these pursuits rarer.

Longitudinal studies of substance use typically come in two forms: 1) studies exclusively of PWUDs (i.e., our team’s data), and 2) studies of a larger population that may also include PWUDs. The latter type is more common as many longitudinal studies use a sample design to be representative of a general population (such as U.S. adults) and are not focused exclusively on PWUDs. Within those studies, they may ask a series of questions about drugs, but PWUDs are not their primary focus. Both the National Longitudinal Study of Youth (NLSY) and the National Study of Adolescent Health (Add Health) are prime examples of these types of projects that follow a representative sample over time and include some questions on substance use. Although lacking a PWUDs-focused sample, the contributions of these types of studies can be substantial. For example, a recent review of Add Health’s contributions to longitudinal substance use research identified over 40 papers on substance use from their general population study [13].

Longitudinal studies that sample exclusively people who use drugs and follow them over time are much rarer but focus on issues that are specific to PWUDs, such as overdose experiences [14], HIV and hepatitis C risk environments [15, 16] and changing patterns of use over time [17]. However, unlike our team’s data, most of these longitudinal studies have been focused in urban areas, especially in the U.S. Compared to PWUDs in the urban, those in rural areas may see higher levels of stimulant-related overdose death [18], different patterns of substance use [19, 20], routes of administration [21], and barriers to treatment [22, 23]. A rare exception is the correlational study by Havens and colleagues focusing on rural Appalachia in the U.S. [24], which studied a more restrictive group of PWUDs that had recently used either meth, cocaine, opioids, or heroin and spanned eight longitudinal waves from 2008 to 2020 at roughly 2 to 3-year time intervals.

#### Modeling substance use behaviors

Prior research on modeling substance use behaviors sought to identify its correlates in hopes of informing healthcare providers, policymakers, and other stakeholders toward more efficient allocation of behavioral interventions for PWUDs with the greatest emerging needs. The studies from this area of research have shown that many individual attributes are associated with substance use behaviors: onset of drug use [12, 25–28], criminal involvement [12, 25, 27, 29, 30], drug treatment [12, 25, 27, 31, 32], engagement in drug-free activities [33], marital status [29], education status [29], employment status [26], adverse childhood experience [29], cessation history [30], psychiatric disorder [34], and family environments [35–37] (e.g., support [26, 38], parenting [39–42], parental knowledge [43], and parental expectations [40]). Unfortunately, most work has been correlational based on traditional statistical methods and thus does not consider models that can (stochastically) forecast or *predict* short-term substance use behaviors.

Toward predicting short-term changes in substance use, rather than seeking inferences about correlates, the main goal is to optimize predictive performance. With model complexity adjustable via regularization while making minimal assumptions about the data-generating systems [44–46], machine learning (ML) has proven to be highly suitable for such goal. ML approaches toward substance use that leverage longitudinal data is an area for analytic growth [47]. A 2020 review [48] on predicting later substance use disorders using longitudinal data noted that they were unaware of any ML approaches for predicting substance use disorders, despite the methodology’s strength in assessing complex relationships between a high number of factors. One recent exception is a study from Sweden that used population registry data to examine the comorbidity of ADHD and substance use among youth [49]. Although population registry data does not exist in the U.S., this Swedish example shows the promise of ML techniques being applied to existing longitudinal data sources. A more recent study conducted in 2022 by Rajapaksha et al. [50] attempted to predict the long-term (in at least a few years) risk of developing marijuana use disorder in adulthood for adolescent or young adult marijuana users using Add Health data, which is not PWUDs-focused as mentioned earlier.

For other types of substance use data, ML has been applied [47, 51] for modeling: alcohol use by adolescents [52], long-term drug use patterns [53], impulsivity in cocaine use [54], treatment success [55], initiation [56], continued misuse [57, 58], and development of SUD [59]. However, all of these studies focus on timescales of years. In practice, shorter timescales within months or even days are more desirable as motivated by their relevance to just-in-time (JIT) substance use interventions [6, 9, 10, 60–62]. An exception is the recent series of work by Lo-Ciganic et al. [63–65] that aims to predict opioid use disorder and overdose among Medicare beneficiaries in the subsequent 3 months after initiation of prescription opioids, which relied exclusively on medical professionals’ diagnoses for identifying/labeling at-risk individuals.

### Data

Our data consists of a sample of 237 (anonymized) persons who used drugs (PWUDs) in the Great Plains of the U.S., who were enrolled through peer-based recruitment under the respondent-driven sampling scheme [66]. Recruitment began using fliers for possible participants who were eligible if they had used an illicit substance other than marijuana in the past seven days and passed a cognitive screening test. Each PWUD then completed a survey on a laptop. Once the survey was complete, each PWUD was given up to five coupons that they could give to people in their social networks who were qualified for the study. For each coupon that was returned by a new eligible PWUD, the recruiter was given $10. Recruitment continued through peer recruitment and walk-ins from posted fliers. The process repeats until the desired sample size is reached.

The sample is divided into two cohorts, “Cohort 1” and “Cohort 2”, which contain longitudinal survey data collected from 35 and 202 PWUDs, respectively, each across two different time periods, to which we will refer as Wave 1 (first time period) and Wave 2 (last time period). Cohort 1 Wave 1 data was collected from October 1, 2019 until March 11, 2020 when COVID-19 suspended human subjects research. Wave 2 of Cohort 1 was collected from October 1 to November 30, 2020. Cohort 2 Wave 1 and Wave 2 data was collected beginning in June 14, 2021 and May 3, 2022, respectively, and has continued to the present time. The two waves within both cohorts are 4–12 months apart, and PWUDs completed the same survey in both waves. All PWUDs were informed with a written consent before filling out the survey. The data is collected under IRB approval. We have gained access to all data under the standard Data Sharing Agreement and protocols.

#### Features

In the survey, each PWUD answered questions regarding their individual attributes (including drug use behavior of various drugs) in a largely self-administered manner. Collectively, the responses to these questions form the features in our data. Due to the confidentiality agreement and IRB approval, we only have access to a subset of features, which are listed in Table 1. The features are categorized into different groups based on the question blocks to which they belong in the survey. Details of the survey questions can be found in S1 File. There are initially 151 features in total. The injection drugs considered are injection heroin, injection opioids, injection meth, injection cocaine, heroin-cocaine speedball, heroin-meth speedball, crack cocaine, and buprenorphine (8 in total); and the non-injection drugs include marijuana, cocaine, Ecstasy, PCP, amphetamines, meth, barbiturates, benzodiazepines, opioids, and heroin (10 in total). We focus on the PWUDs’ substance use, which is measured on an ordinal scale (1–8) of {never, less than once a month, once a month, once a week, 2–6 times a week, once a day, 2–3 times a day, 4 or more times a day} for non-injection drugs, and on a similar ordinal scale but with one extra category of “never injected” added prior to “never” for injection drugs (to be discussed shortly).

**Table 1:** Individual features included in our survey data.

Individual Attributes	Summary of Information Collected
Community	• community the participant lives in (e.g., years, satisfaction, feeling, and preference)
Social Support	• people the individual can confide in, obtain emotional support, and provide financial assistance
Drug Accessibility & Use Patterns	• accessibility and perceived availability of various substances, and time of day of drug usage
Drug Overdose	• experiences of overdoses (e.g., personal overdose history and other overdose users)
Substance Use Treatment	• individual participation in alcohol and drug treatment programs (e.g., outpatient and detox)
Adverse Childhood Experiences	• adverse childhood experiences (e.g., verbal humiliation), family closeness, and family issues
Criminal Justice Involvement	• individual experiences/concerns with police and incarceration including concerns with police
Tobacco Use	• regular use, first starting smoking age, frequency of smoking cigarettes, e-cigarettes, and tobacco
Alcohol Use	• age of first started drinking and frequency of drinking is collected
Injection Drug Use	• first injection age, various substance usages past 6 months, injection locations, and needle sharing
Non-injection Drug Use	• various substance usage past 6 months and locations of usage
Demographics	• age, marital status, education, current employment status, religion, influence of religious or spiritual beliefs, ever been homeless, total household income

#### Data preprocessing

Due to the large number of features (151) relative to the sample size of either Cohort 1 and Cohort 2 and that the distributions for most features from the two cohorts are similar, we first combine PWUDs from both in order to get a larger sample size of 35 + 202 = 237. We then reduce the dimension of our dataset by discarding some uninformative individual features:

- PWUDs’ and interviewers’ ID tags, which are generated to prevent duplication only.
- All text features. The first type includes other drugs (injection and non-injection) used that are not listed. Very few PWUDs (less than 20%) reported using non-listed drugs, and nearly all of their inputs are, in one way or another, already listed drugs. For instance, “Molly” is another name for Ecstasy/MDMA, “Xanax” is a subtype of benzodiazepines, and “Suboxone” is a combination of buprenorphine and other drugs. Other text features are drug-using locations, in which there are too many non-standardized inputs e.g., home, car, park, alley, “with friends”, “at work”, etc. with respect to the sample size. Therefore, we omit all information from these text features in our analyses.

**Fig 1:**
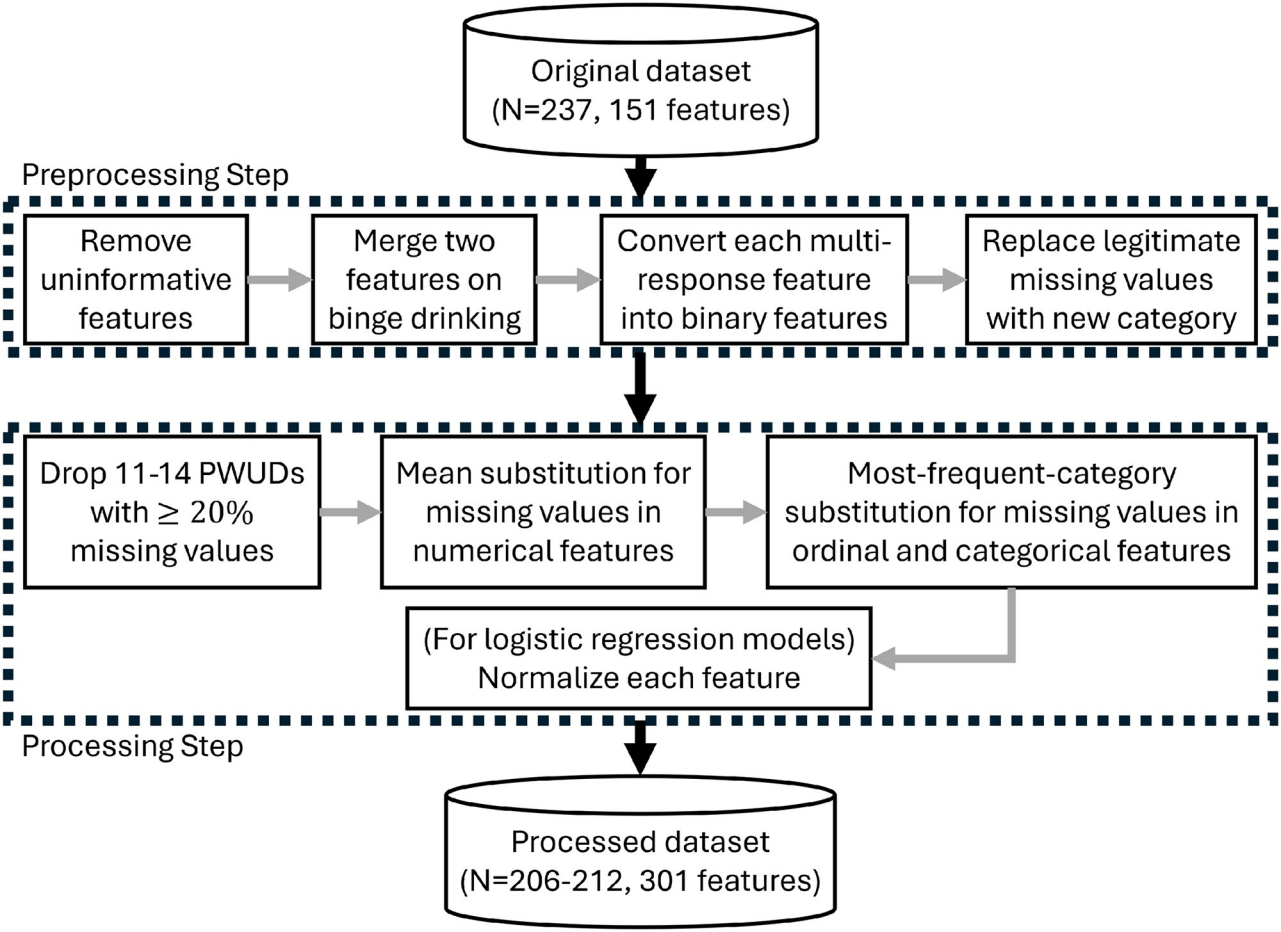
Flowchart outlining our data preprocessing and data processing methodology.

Given the nature of survey data, we preprocess some of the raw features in our dataset in order to facilitate the analyses at the population level. Fig 1 outlines the data preprocessing as well as the data processing steps that will be described shortly.

- Two features linked to the same question asking how often PWUDs have binge drinking i.e., *n* or more alcoholic drinks in one sitting (*n* = 4 and 5 for female and male, respectively) are merged into one since the filled entries across these two features are mutually exclusive.
- The features associated with multiple/check-all-that-apply responses are converted separately into binary features. For example, the question asking which drug(s) (out of the *k* listed drugs) are used in the morning is converted into *k* binary features, each indicating whether PWUDs used drug *i* or not.
- Missing values within our dataset are classified as (1) *legitimate* [67], due to *skip logic* from survey design, in which PWUDs are allowed to skip to certain questions depending on how they responded in the previous ones; and (2) *non-legitimate* or “actual missing data” otherwise. We only deal with the former in the current preprocessing procedure as it constitutes a substantial portion of the data (10% of the entire dataset). In particular, for legitimate missing values occurring in some questions, we added a new category for the corresponding feature. If the feature is ordinal, we choose an appropriate numerical value to maintain the hierarchy and then assign it to the missing values. For example, in the question asking the frequency of injection heroin within the last 6 months (choose one out of the eight categories: (1) “never” ≺ (2) “less than once a month” ≺ (3) “once a month” ≺ … ≺ (8) “4 or more times a day”), if some PWUDs skipped because they responded earlier that they have never injected any drug before at any point, then we categorize them as “never injected (any drug before)” and hence this new category would precede “never” in the order. If the missingness is otherwise non-legitimate, we follow the imputation scheme discussed below in our data processing step. For the analyses at the population level, we do not take into account entries having non-legitimate missing values such as when counting PWUDs exhibiting an increase/decrease in usage of a certain drug.

### Statistical analysis

The goal of the analysis is to study the predictive individual attributes that can predict the short-term changes in substance use for PWUDs within the population. More specifically, we investigate whether simple machine learning models (i.e., logistic regression and decision trees) can address the following two main short-term predictive questions: (i) predicting which drug(s) a PWUD would (likely) use and (ii) predicting which drug(s) a PWUD would (likely) increase usage. Predictive question (i) would inform healthcare providers on determining appropriate resources to allocate to PWUDs; and predictive question (ii) would enable the identification of at-risk PWUDs (i.e., increased drug usage).

#### Problem formulation

For each of our predictive questions, we can naturally cast it as a multilabel classification problem. More specifically, for predictive question (i), the corresponding multilabel classification problem aims to identify a list of drugs (based on a probability distribution over the considered drugs) that PWUDs will likely use within 12 months. To infer PWUDs’ probabilities of using *n* different drugs, we break the problem down into *n* separate binary classification problems, one for each drug. In each binary classification problem for a particular drug, PWUDs that used it within the next 12 months belong to the positive class (i.e., having positive label ‘1’) while those that did not use the particular drug belong to the negative class (i.e., having negative label ‘0’). The problem then seeks to predict the label of 1 or 0 for each PWUD.

For predictive question (ii), the problem aims to identify a list of drugs that PWUDs will likely increase their use within 12 months. To infer a PWUD’s probabilities of increasing usage of different drugs, similar to the first question, we train a separate binary classification model for each drug wherein PWUDs exhibiting an increase and non-increase (i.e., decrease or unchanged) in the usage of this drug belong to the positive and negative class, respectively.

In both problems, once we train all separate binary classifiers for all considered drugs, the final combined model predicts all labels (use drug *x* or not for the first problem and increase or non-increase in *x*’s usage for the second) for some unseen sample of PWUDs. This approach is also known as the *binary relevance* method [68]. We chose this formulation despite its simplicity compared to more advanced methods such as classifier chains [68] and ensemble method [69] because our goal is to ensure that our results have a widespread impact and are accessible to key players such as healthcare providers, mental health professionals, and policymakers.

**Fig 2:**
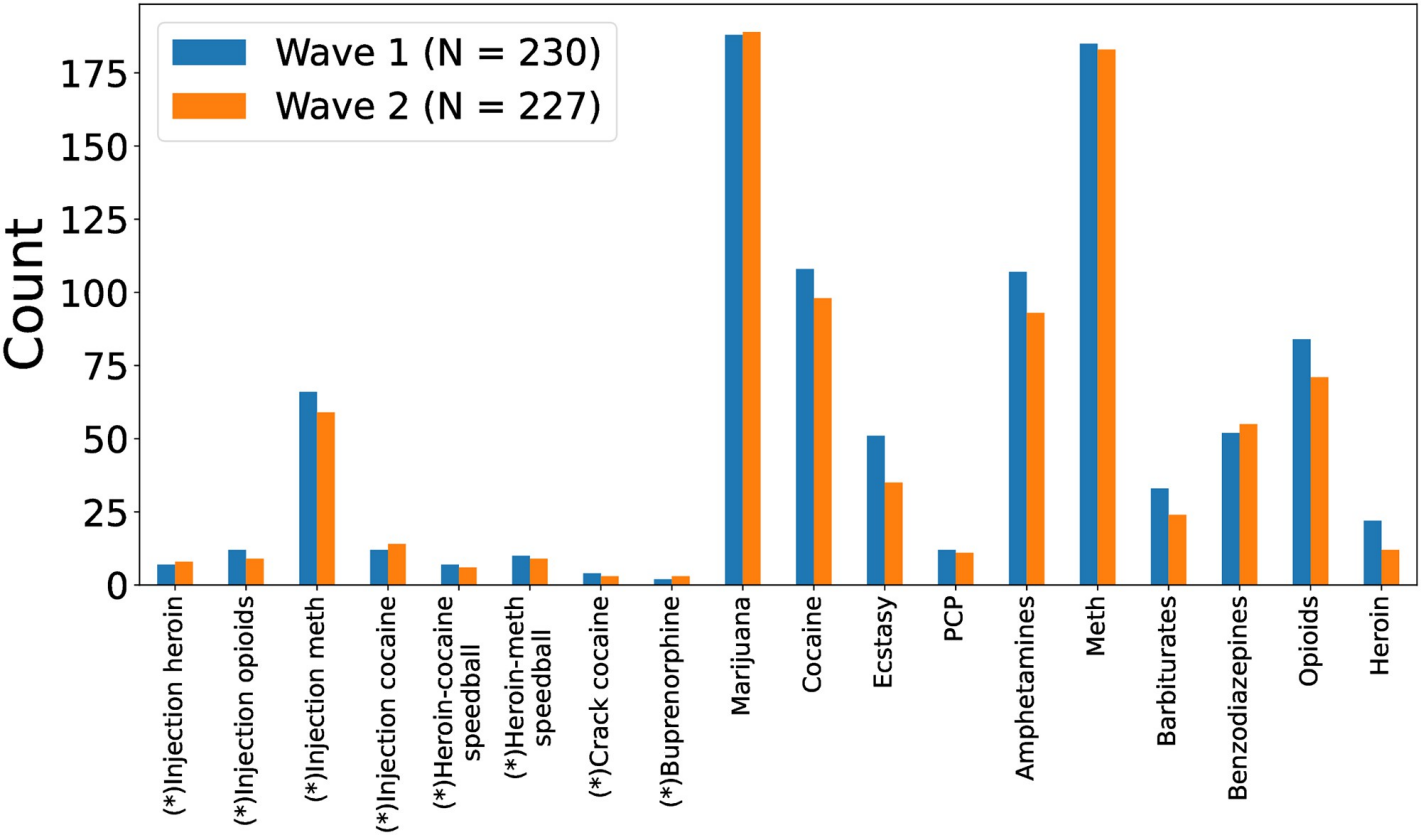
Bar chart showing the prevalence of each considered drug at Wave 1 versus Wave 2. Injection drugs are denoted by an asterisk (*). There are *N* = 230 and 227 PWUDs that used at least one drug at Wave 1 and Wave 2, respectively.

In terms of drug choice, we focus on the most prevalent drugs (within our data, see Fig 2) that have at least 25% of PWUDs that changed their usage and at least 10% exhibiting an increase within the next 12 months (see Table 3), which includes marijuana, meth, amphetamines, cocaine, opioids, benzodiazepines, and injection meth. The experimental results for the last three drugs can be found in S3 and S10 Tables.

In all binary classification problems for both predictive questions, we follow the same considerations as presented below.

#### Classifiers

We consider two binary classifiers: logistic regression (LG) and decision tree (DT) i.e., **C**lassification **a**nd **R**egression **T**rees (CART) [70], which were implemented using Scikit-learn v1.0.2 library [71]. Apart from their interpretability, both can also output class posterior probabilities (Decision tree classifier can return posterior probabilities from the percentage of the majority label at the leaves via Platt scaling.), which can be used for quantitatively evaluating how likely a given PWUD would use or would increase usage of a certain drug.

Since we are working with high-dimension low-sample-size (HDLSS) data, we perform elastic-net regularization [72] and tree pruning to prevent the LG and DT classifiers from overfitting our model, respectively. The regularization strength (stronger yields simpler models) can be controlled via a set of so-called *hyperparameters*, which are parameters whose values are manually pre-set and fixed throughout the training process. We define the search space to identify the optimal hyperparameters during cross-validation (to be discussed shortly) as follows:

- For LG, we tune the regularization parameter *C* = 10^*k*^,
  where *k* ∈ {−3, −2, −1, 0, 1, 2, 3}, and the elastic-net mixing parameter *ρ* of range {0, 0.1, 0.2, …, 0.8, 0.9, 1}. When *ρ* = 1 and 0, the regularization is equivalent to *ℓ*_1_ (Lasso) and *ℓ*_2_ (Ridge), respectively.
- For DT, we tune the maximum depth of tree ∈ {1, 2, …, 9, 10} and the minimum number of samples required to split an internal (i.e., non-leaf) node ∈ {5, 10, 15, 20, 25}, which effectively control the tree’s complexity.

#### Data processing

In order to make our data easier to digest by the ML classifiers, we further process our data as follows. After filling in values for legitimate missing data, the remaining non-legitimate missing entries (less than 5% of the entire dataset) undergo the following imputation steps (Some survey questions provide the option “don’t know” or “unsure”. We treat responses from PWUDs falling into this category as non-legitimate missing values as it might disrupt the hierarchy of some ordinal features e.g., those having possible values of {very hard, hard, neither hard nor easy, easy, very easy, don’t know}.). First, we drop PWUDs that have 20% or greater missing *raw individual* features: 13 for marijuana, 14 for meth, 11 for amphetamines, and 14 for cocaine. Afterwards,

- for numerical features, we apply the commonly used *mean substitution* imputation technique by replacing the missing entries within a certain feature with the mean of the available values from that feature.
- for ordinal features, after label encoding, we fill in the category that occurs most frequently within that feature, which is the categorical equivalence of the mean substitution [73]. This is also applied to the binary features from the check-all-that-apply questions.
- for categorical or nominal features, *before* one-hot encoding, we also assign its most frequent category to the respective missing entries, *then* perform one-hot encoding afterward. The reason for this is to avoid cases where the binary vector representing the category chosen by PWUDs has more than one entry with a value of 1 as a result of the imputation. As an example, consider the feature on whether the PWUD had suffered drug overdose in the past 6 months, where there are three possible choices/categories: “No”, “Yes”, or “Not applicable” (if they responded earlier that they never suffered drug overdose in their life). Each category is then represented as a vector of [1, 0, 0], [0, 1, 0], [0, 0, 1], respectively. Assuming a PWUD has this feature missing, and the most frequent values for the three resulting binary features (which are determined independently for each) are 1 (“No”), 0 (non-“Yes”), and 1 (“Not applicable”), then it makes little sense for this PWUD to take the value of [1, 0, 1] for this feature since they cannot choose both “No” and “Not applicable” at the same time.

We chose these simple imputation techniques after empirically considering more advanced ones such as maximum likelihood [74, 75] and multiple imputation with random forest [76] and observing negligible differences in the predictive performance of the resulting models.

Additionally, for any model using (regularized) LG classifier, we want all features to be penalized equally regardless of the range of its values. Therefore, we ensure all features are on the same scale using the standard *min-max normalization* method [77], which is non-parametric i.e., distribution-free and can deal better with outliers, on each feature.

#### Performance measures

We evaluate the performance of the trained models on some test set by computing the **A**rea **u**nder the **R**eceiver **O**perating **C**haracteristic curve (AUROC) and **A**rea **u**nder the **P**recision-**R**ecall curve (AUPR), which are less sensitive to class imbalance [78]. The AUROC is determined by first constructing the ROC—plot of true positive rate versus false positive rate evaluated on the test set at different threshold settings, then computing the area under this ROC. Here, “threshold” refers to a value in the range of [0, 1] at or above which a predicted probability from the model is converted to a class label of 1, otherwise to a class label of 0. We can interpret the AUROC as the probability that a randomly chosen PWUD with a positive label (used certain drug for (i) or increased its usage for (ii) within the next 12 months) ranks above a randomly chosen PWUD with a negative label. The model with a high AUROC (approaching 1) is therefore able to distinguish the classes effectively; otherwise, an AUROC close to 0.5 (baseline) indicates its poor classification performance i.e., making completely random predictions. The AUPR is captured by ∑_*k*_(*R*_*k*_ − *R*_*k*−1_)*P*_*k*_ where *P*_*k*_ and *R*_*k*_ are the precision and recall evaluated on the test set at the *k*th threshold (increasing in the range of [0, 1]). This expression is also known as “average precision”, with weight as the increase in recall from the previous threshold. The model with a high AUPR (upper bounded by 1) can correctly classify most positive PWUDs without incorrectly classifying too many negative PWUDs as positive; whereas the model that makes random predictions has an AUPR close to the proportion of positively-labeled PWUDs in the entire dataset (baseline). Compared to AUROC that captures the overall performance of a classifier at various probability thresholds, AUPR puts more emphasis on correct classification of the positive class, which is particularly useful for severe class imbalance settings. Thus, as shown in our results, the model that returns the highest score for one measure might not achieve so for the other measure.

#### Model validation

To minimize bias of the performance estimates (i.e., optimism in the values of the above-considered measures) while validating each model, we employ *nested cross validation* (CV) technique as recommended by [79–81] for HDLSS settings. The nested CV has an inner loop, which is responsible for model development i.e., hyperparameter tuning, nested inside an outer loop, which is used for estimating the generalization performance. For our setting, the inner loop has 10 folds, and the outer loop comprises an 80: 20 train-test split of our data, which is repeated/shuffled for 100 iterations. More specifically, for each train-test split, we further divide the training set (80% of the complete dataset) into 10 smaller sets or “folds”; for each of these 10 folds, (i) a model is trained using 10 − 1 = 9 of the folds as training data, then (ii) the resulting model is validated on the remaining part of the data (the left-out fold); the set of hyperparameters from the model that yields the highest accuracy is kept to train a larger model using all 10 folds as training data (i.e., the original training set), which is validated on the held-out data of the current split (20% of complete dataset). Finally, we calculate the average and the corresponding standard error of the scores over 100 iterations for each performance metric, where the latter is defined as

$$\begin{array}{c} \frac{1}{\sqrt{N}}\sqrt{\frac{\sum_{i=1}^{N}{\left(a_{i}-\bar{a}\right)}^{2}}{N-1}}, \end{array}$$

with *a*_*i*_ the recorded metric e.g., at the *i*th split and $\bar{a}$ the average across all *N* = 100 iterations. We specify a random seed before generating each train-test split to guarantee reproducibility and consistency across the experiments.

#### Feature selection

Due to the greater number of features (301) with respect to the currently available sample size (*N* = 206 to 212 after all data processing), we carefully perform feature selection before training our models, which is validated using the aforementioned nested CV technique. We consider the following conventional heuristics:

- Selecting top-*k* features having the highest mutual information [82, 83] with the class labels, where *k* ∈ [1, 20] is included as an additional hyperparameter and tuned during the inner CV loop.
- Selecting features having non-zero permutation-based feature importance with random forest [84]. Essentially, a random forest of (100) decision trees is trained and the importance of each feature is computed afterward based on how much the model depends on it while making predictions (refer to [84] for technical details). The features having non-zero importance scores (generally less than 20) are then selected.
- Forward sequential feature selection [85], where the features are iteratively added to the set of selected features in a greedy fashion. Initially, the algorithm starts with zero features and finds the one best feature subject to the cross-validated accuracy (which is returned from the trained model having this single feature as input) within the inner CV loop. The procedure then repeats by iteratively adding new best features and stops when the maximum number of selected features is reached, which we set to 20 for consistency with the above two methods. Finally, the combination between 1 and 20 features that score highest in the inner CV is selected. Note that hyperparameter optimization is performed during the training of *each* model and hence the optimal set of hyperparameters is determined independently for a given combination of features.
- Multi-objective genetic algorithm [86], where the objectives for the fitness function are: (1) maximizing the mean (inner) CV accuracy, (2) minimizing the number of features selected, and (3) minimizing the standard deviation of the accuracy across the inner CV folds. The subset of features (of any size) that is Pareto optimal (i.e., reaches a point where none of the three objectives can be improved without sacrificing at least one of the two other objectives) is selected.

In addition to the aforementioned “black-box” methods commonly used in ML literature, we consider two transparent approaches where we:

- Group features according to the question block to which they belong (as shown in Table 1) and manually combine these groups based on their relevancy:
  - Information on PWUDs’ social support, community, and demographics (SCDM);
  - Substance use behaviors (SUB): tobacco, alcohol, injection drug, and non-injection drug use, which include information on four more substances (cigarette, electronic cigarette i.e., vape, smokeless tobacco, and alcohol) in addition to the considered 18 drugs; and
  - Traumatic experiences (TREX): drug overdose, adverse childhood experiences, and criminal justice involvement.
- Perform bivariate analyses beforehand to identify features that are highly correlated with the target variable. In particular, we first conduct a chi-square test of independence [87] for ordinal and categorical features and point-biserial correlation coefficient [88] for numerical features to examine its correlation with the binary labels. All analyses conform to the conventional significance level of *α* = 0.05 and were conducted using SciPy v1.7.3 library [89]. Then, we combine the top *k* correlated features exhaustively for a total of 2^*k*^ − 1 combinations of sizes 1, 2, …, *k* − 1, *k*. Due to the sheer size of the power set given our limited computing power, we restrict *k* to be no more than 10.

## Results

### Objective 1: Analyzing short-term substance use behaviors of PWUDs at the population level

For our first objective, we analyze the short-term substance use behaviors from PWUDs at the population level by first providing some basic statistics of our data, specifically the most prevalent drugs, the number of distinct drugs used by PWUDs in general, and the combinations of drugs that were commonly used in conjunction, then examining the changes (i.e., increase and decrease in usage of each drug across the two longitudinal waves). Note that we only consider the 18 aforementioned injection and non-injection drugs, which are categorized as illicit drugs (or partially illicit as for marijuana) in the U.S., and therefore exclude tobacco and alcohol.

#### Short-term substance use statistics in two waves

To help visualize how prevalent each drug is, Fig 2 shows the bar chart for counting PWUDs that used certain drugs by wave. Although the relative prevalence of different drugs stays the same across the two waves—marijuana, meth, amphetamines, and cocaine are most prevalent in descending order whereas injection drugs (denoted by an asterisk) are much less prevalent than non-injection drugs except for injection meth—we observe a small drop in the count at Wave 2 with respect to the count at Wave 1 for most drugs.

**Fig 3:**
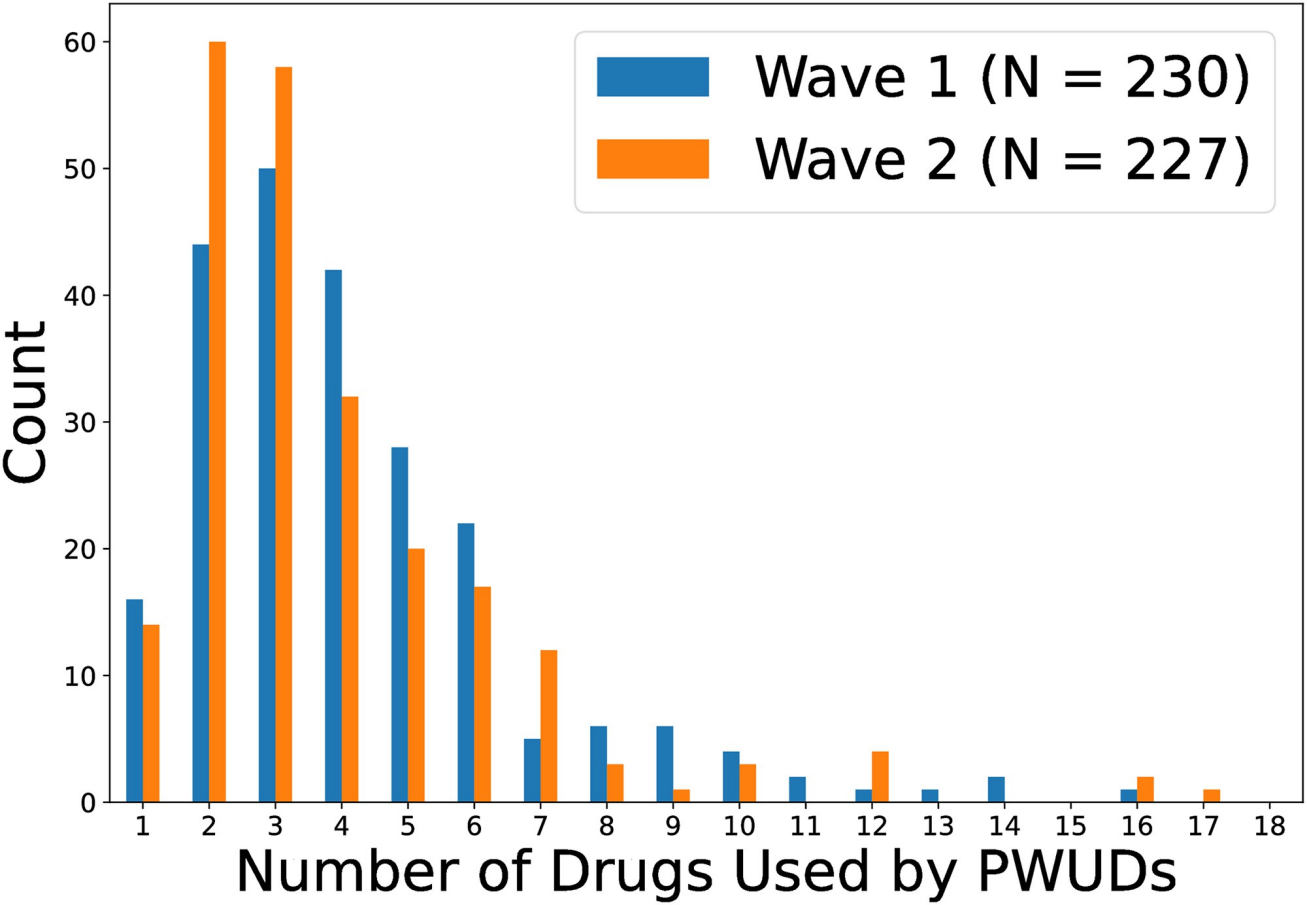
Histogram of number of drugs used by PWUDs at Wave 1 versus at Wave 2.

As PWUDs likely use more than one drug [90], we examine the number of drugs used by them across different waves. Fig 3 shows the histogram of the number of drugs used by waves. We observe a gamma distribution in both waves, though the one at Wave 2 seems to exhibit higher skewness. In particular, PWUDs generally used fewer drugs at Wave 2 versus at Wave 1, with 2 different drugs used compared to 3. The outliers from Wave 2 (compared to Wave 1) are also more extreme with up to 17 drugs used compared to 16.

Regarding which drugs are most commonly used together, Table 2 lists the top 5 prevalent combinations of drugs used concurrently at each wave, which were observed in ∼30% of PWUDs in total. Either marijuana or meth is present in all of the listed combinations, and not surprisingly, both combined are the most prevalent, which was observed in ∼10% of PWUDs at both waves. The next four most prevalent combinations across the two waves include amphetamines, cocaine, and/or injection meth. Our follow-up analyses (summarized in S1 Table) showed that within 12 months from Wave 1 to Wave 2, more than 80% of PWUDs changed their combination (by at least one drug). There were also fewer unique drug combinations at Wave 2, with 99 (62 are exclusive to one PWUD alone) compared to 113 drug combinations (77 are exclusive) at Wave 1. Half of all PWUDs had drug combinations that were among the top 14 prevalent at Wave 2 versus among the top 17 at Wave 1 (refer to S2 Table).

**Table 2:** Top-5 prevalent combinations of drugs used by PWUDs. The respective counts are shown in parentheses and the sum of counts against the total number of PWUDs that used at least one drug is shown in the bottom row.

Wave 1	Wave 2
Marijuana + Meth (20)	Marijuana + Meth (24)
Marijuana + Meth + Inj. meth (16)	Marijuana + Meth + Cocaine (16)
Meth (alone) (11)	Marijuana + Meth + Amphetamines (12)
Marijuana + Meth + Amphetamines + Cocaine (10)	Marijuana + Meth + Inj. meth (11)
Marijuana + Meth + Cocaine (10)	Marijuana + Cocaine (9)
67/230	72/227

#### Extent of changes in substance usage of PWUDs

Our earlier observations strongly indicate a certain degree of change in short-term substance use behaviors from PWUDs. We now analyze how many and to what extent PWUDs increased and/or decreased their usage (Those who reported a decrease in drug usage during the time of interviewing at Wave 2 might exhibit an increase at any point later on.).

#### Percentage and variation of changes in substance usage

For each drug, we investigate the percentage of PWUDs that exhibit an increase/decrease within the span of 12 months. Table 3 ranks the drugs by percent of PWUDs with an increase with respect to the sample size *N*, in descending order. The corresponding percent of decrease and overall percent of change are also shown in the rightmost columns. Note that *N* varies for each drug due to the missing information of usage for the respective drug at either wave from some PWUDs (7 to 28) during the data collection phase. The associated count is recorded in parentheses. We observed that in general, PWUDs change their usage of non-injection drugs more often than their usage of injection drugs (marked with an asterisk).

**Table 3:** Drugs sorted by descending percent of PWUDs with an *increase* within 12 months (with respect to *N*, the total number of available PWUDs). The discrepancy in *N* between the drugs is due to the missing usage information for certain drugs at either wave. Injection drugs are denoted by an asterisk (*).

Drug	*N*	% Increase (Count)	% Decrease (Count)	% Change
Marijuana	225	29.5 (70)	33.8 (80)	66.7
Meth	220	26.6 (63)	31.6 (75)	62.7
Amphetamines	219	18.1 (43)	30.8 (73)	53.0
Cocaine	225	15.2 (36)	27.4 (65)	44.9
Opioids	221	14.3 (34)	20.3 (48)	37.1
Benzodiazepines	222	11.4 (27)	12.7 (30)	25.7
Injection meth*	226	10.1 (24)	18.6 (44)	30.1
Injection cocaine*	229	7.6 (18)	9.3 (22)	17.5
Ecstasy	209	7.6 (18)	14.3 (34)	24.9
Barbiturates	219	7.2 (17)	10.5 (25)	19.2
Injection heroin*	230	5.5 (13)	7.2 (17)	13.0
Injection opioids*	225	5.5 (13)	8.4 (20)	14.7
Heroin-meth speedball*	226	5.5 (13)	8.0 (19)	14.2
Heroin-cocaine speedball*	229	5.1 (12)	7.6 (18)	13.1
Crack cocaine*	227	4.2 (10)	6.3 (15)	11.0
Buprenorphine*	225	4.2 (10)	5.9 (14)	10.7
PCP	223	2.5 (6)	3.0 (7)	5.8
Heroin	220	2.1 (5)	7.2 (17)	10.0

**Fig 4:**
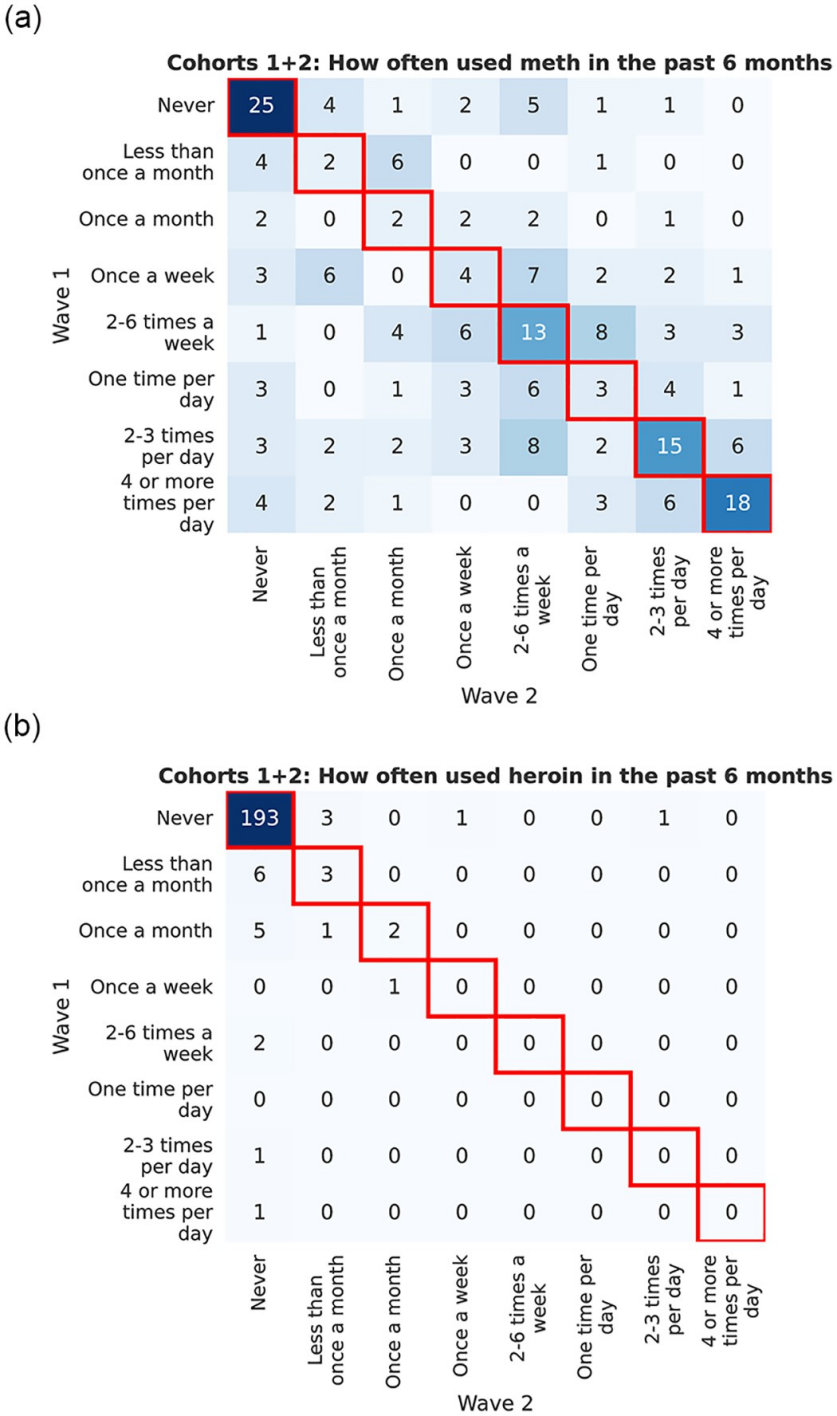
Change in usage of (a) meth and of (b) heroin across two waves. The upper triangle of the heatmap exhibits increased usage within the 12-month period.

We further illustrate the changes in usage for two drugs of choice, meth and heroin (respectively exhibiting high and low % change) using heatmaps in Fig 4. In each heatmap, the y-axis shows drug use frequency in ordinal scale (incrementing from top to bottom) at Wave 1, and the x-axis similarly shows usage (incrementing from left to right) at Wave 2. A cell at row *y* = *i* and column *x* = *j* displays the number of PWUDs with usage *i* of certain drugs at Wave 1 and usage *j* at Wave 2, with darker colors associated with larger counts. For either drug, since both waves share the exact same set of ordered usage categories, the heatmaps can be treated as square matrices, where PWUDs with increased/decreased usage lie in the upper/lower triangle (i.e., above/below the diagonal highlighted in red). At any current usage of meth i.e., row-wise, we see that the number of PWUDs with an increase versus the number of non-increases are comparable. As an example, among the 25 PWUDs that used meth once a week at Wave 1, 12 increased their usage at Wave 2 while 13 did not. In contrast, none of the PWUDs that used heroin at Wave 1 had an increase at Wave 2. For other drugs, the pattern of changes lies somewhere in between. For example, for cocaine, which has a lower variation of changes than meth, there are fewer PWUDs with an increase in cocaine than those with a non-increase at any given current usage.

#### Distribution of the extent of changes in substance usage

**Fig 5:**
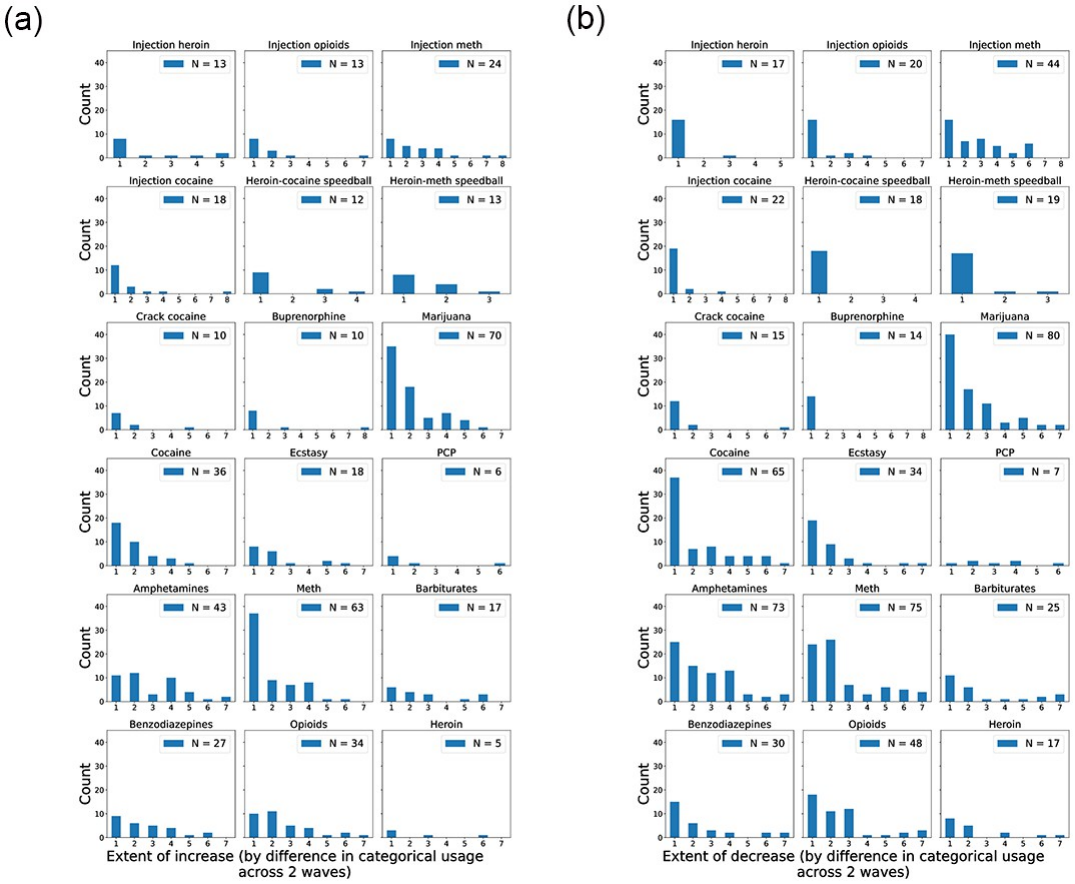
Histograms of the extent of the (a) increase and (b) decrease by the difference in categorical usage within 12 months for all drugs.

For each drug, we look more closely at the distribution of how much PWUDs increased and decreased their use in terms of the difference in categorical usage in Fig 5(a) and 5(b), respectively. For instance, given the usage ordering of {“never” ≺ “less than once a month” ≺ “once a month” ≺ “once a week” ≺ “2–6 times a week” ≺ “once a day” ≺ “2–3 times a day” ≺ “4 or more times a day”} from the survey, if some PWUDs reported using certain drug with usage *i* = “once a month” at Wave 1 up to *j* = “2–6 times a week” at Wave 2, the extent of increase for this drug is quantified as 2 because *j* is two categories above *i* (Recall that injection drugs come with one extra category.). The same applies to decreases. As seen in the figure, interestingly, the histograms for the majority of drugs, e.g., marijuana, cocaine, and meth, exhibit a power-law distribution, which means most PWUDs did not drastically increase/decrease their usage.

#### Distribution of the numbers of substance use changes

**Fig 6:**
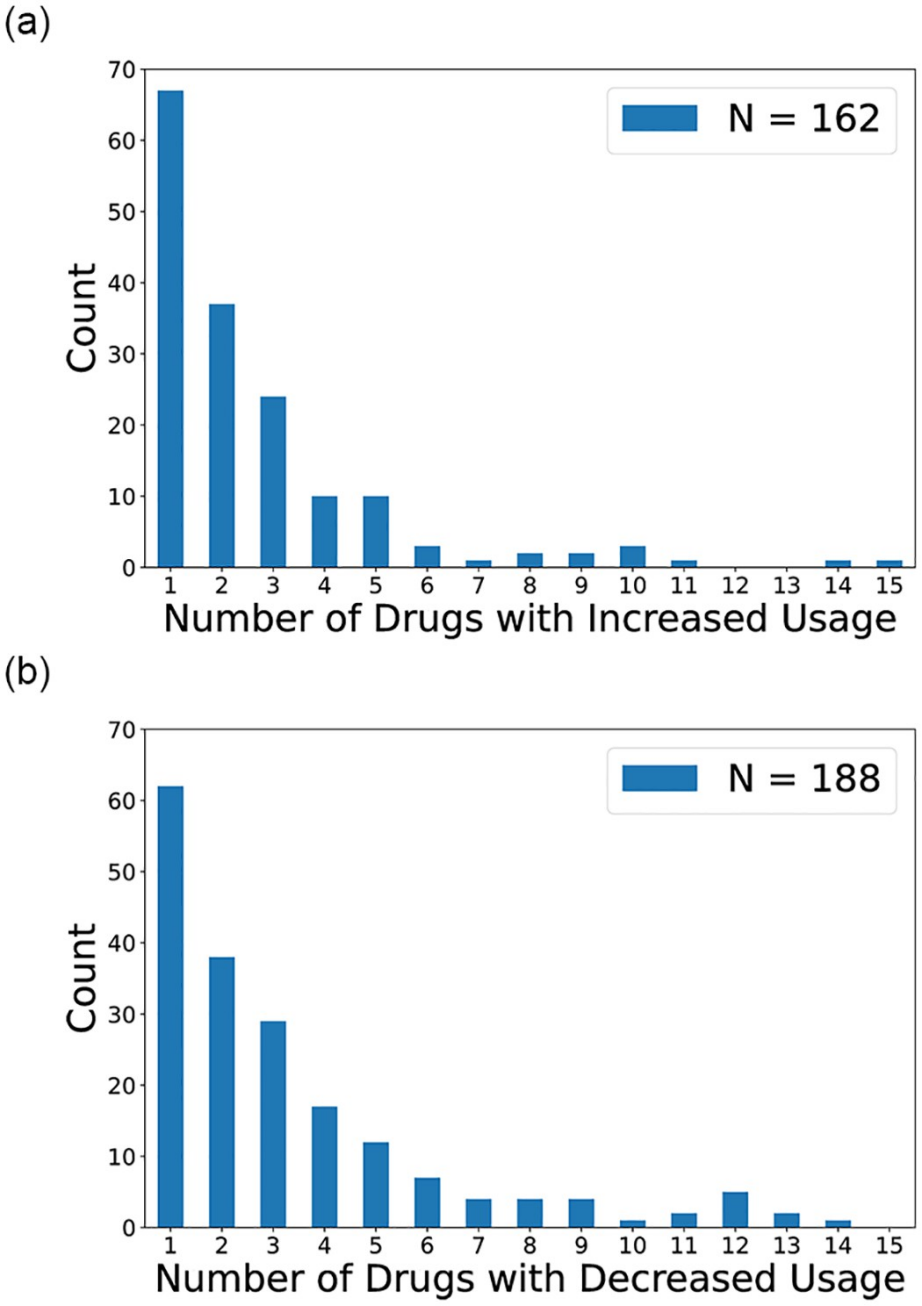
Histogram of number of drugs with (a) increased and (b) decreased usage within 12 months. There are 162 out of 237 PWUDs with an increase and 188 with a decrease in usage of at least one drug.

Among PWUDs that increased/decreased usage of any drug, we also examined collectively how many drugs they increased and decreased in Fig 6(a) and 6(b), respectively. The associated histograms also exhibit a power-law distribution, in which nearly 80% of PWUDs increased usage while nearly 70% decreased usage of 3 or fewer drugs.

### Objective 2: Predicting short-term substance use behaviors of PWUDs at the individual level

#### Predicting drugs likely used in the next 12 months

**Fig 7:**
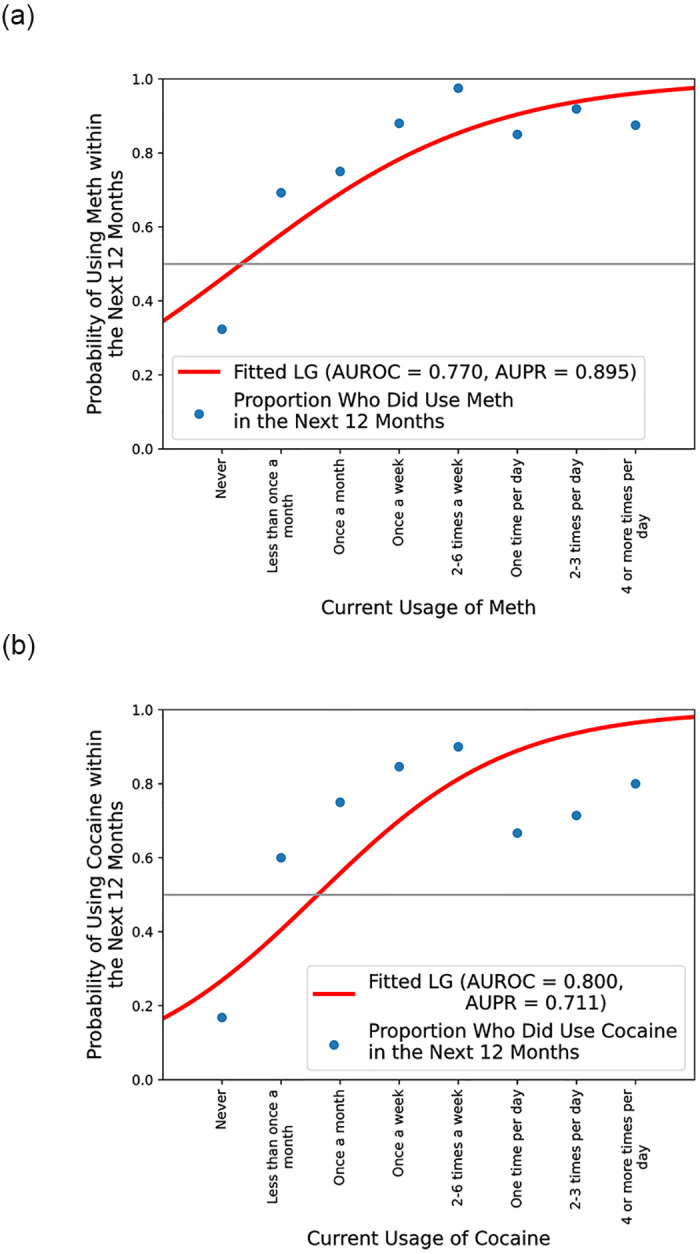
Fitted logistic curve for the LG model using only current usage of (a) meth and (b) cocaine as input. The dots mark the proportion of PWUDs that did use meth/cocaine within the next 12 months given the corresponding current usage.

Besides the baselines of the evaluation metrics, we additionally train a simple baseline model for each drug using the single feature associated with the current usage of the corresponding drug as input. This would give us a proper standard against which more complex models could be benchmarked. Table 4 shows the decent performance of these baseline models relative to the baselines for both AUROC and AUPR (top and bottom of each cell, respectively). For all four considered drugs, the baseline LG model seems to perform better than its DT counterpart, with + 2.96% to + 6.93% higher AUROC and + 1.82% to + 12.8% higher AUPR. The resulting decision boundary for the LG model for meth and cocaine as examples can be seen in Fig 7, which effectively captures the (dotted) factual proportion of PWUDs that used meth within the next 12 months under certain current usage.

**Table 4:** AUROC (top of cell) and AUPR (bottom of cell) measures, averaged over 100 train-test splits, of logistic regression (LG) and decision tree (DT) models for predicting whether PWUDs would use a certain drug within the next 12 months using different feature selection methods. The highest scores are bolded.

Methods	Marijuana		Meth		Amphetamines		Cocaine	
Classifiers	LG	DT	LG	DT	LG	DT	LG	DT
Baseline	0.500		0.500		0.500		0.500	
	0.816		0.794		0.421		0.422	
Current Usage as Lone Predictor	0.787 ± 0.009	0.736 ± 0.009	0.770 ± 0.010	0.742 ± 0.008	0.722 ± 0.006	0.677 ± 0.007	0.800 ± 0.006	0.777 ± 0.006
	0.924 ± 0.004	0.897 ± 0.003	0.895 ± 0.005	0.879 ± 0.004	0.642 ± 0.008	0.569 ± 0.007	0.711 ± 0.008	0.652 ± 0.006
Top *k* Mutual Information	0.756 ± 0.009	0.699 ± 0.011	0.774 ± 0.009	0.738 ± 0.009	0.707 ± 0.007	0.630 ± 0.007	0.767 ± 0.008	0.766 ± 0.006
	0.922 ± 0.004	0.887 ± 0.004	0.901 ± 0.005	0.880 ± 0.004	0.654 ± 0.008	0.536 ± 0.006	0.715 ± 0.009	0.652 ± 0.007
Random Forest	0.715 ± 0.013	0.689 ± 0.013	0.755 ± 0.012	0.697 ± 0.010	0.666 ± 0.010	0.625 ± 0.008	0.741 ± 0.008	0.770 ± 0.007
	0.913 ± 0.005	0.884 ± 0.005	0.902 ± 0.006	0.864 ± 0.004	0.625 ± 0.010	0.528 ± 0.006	0.696 ± 0.008	0.649 ± 0.007
Forward Selection	0.771 ± 0.008	0.548 ± 0.010	0.747 ± 0.010	0.682 ± 0.011	0.685 ± 0.007	0.556 ± 0.010	0.746 ± 0.007	0.700 ± 0.008
	0.932 ± 0.003	0.835 ± 0.004	0.902 ± 0.005	0.861 ± 0.005	0.640 ± 0.008	0.506 ± 0.008	0.698 ± 0.008	0.619 ± 0.008
Genetic Algorithm	0.755 ± 0.008	0.602 ± 0.012	0.736 ± 0.009	0.631 ± 0.011	0.641 ± 0.007	0.564 ± 0.008	0.708 ± 0.008	0.652 ± 0.008
	0.934 ± 0.003	0.853 ± 0.004	0.896 ± 0.005	0.841 ± 0.004	0.594 ± 0.007	0.482 ± 0.006	0.676 ± 0.009	0.555 ± 0.007
Manual Grouping	0.770 ± 0.010	0.726 ± 0.009	0.766 ± 0.010	0.721 ± 0.009	0.725 ± 0.007	0.648 ± 0.006	0.759 ± 0.008	0.776 ± 0.006
	0.927 ± 0.004	0.896 ± 0.003	0.910 ± 0.005	0.872 ± 0.003	0.679 ± 0.008	0.543 ± 0.006	0.715 ± 0.009	0.651 ± 0.007
Top-*k* Correlated	**0.814** ± 0.008	**0.771** ± 0.009	**0.824** ± 0.009	**0.829** ± 0.007	**0.756** ± 0.006	**0.706** ± 0.008	**0.823** ± 0.007	**0.804** ± 0.006
	**0.947** ± 0.003	**0.916** ± 0.004	**0.929** ± 0.004	**0.930** ± 0.003	**0.729** ± 0.007	**0.639** ± 0.008	**0.769** ± 0.009	**0.702** ± 0.008

The remainder of Table 4 shows the performance of the LG and DT models for predicting whether PWUDs would use a certain drug within the next 12 months using the various feature selection methods. First, the models built from the optimal combination of the top *k* correlated features return the highest scores for both AUROC and AUPR. Compared to the baseline LG model using current usage alone as a predictor (which is better than its DT counterpart as discussed earlier), AUROC is improved by 3.43%, 7.66%, 4.71%, 2.88%, and AUPR by 2.49%, 3.91%, 13.6%, 8.16% for prediction of marijuana, meth, amphetamines, and cocaine, respectively.

The models returned from other feature selection methods do not seem to consistently perform better than the baseline models, and even if they do, the improvement is very small (<1%). Among these other methods, despite its straightforwardness, the manual grouping of features generally yields models having the best results, with both AUROC and AUPR comparable to those from the baseline models. We take a closer look at the best manually grouped combinations in Table 5, in which the features capturing non-injection drug use (12 in total) are the most useful in general.

**Table 5:** Partial breakdown of the manual grouping feature selection method. Only the combinations that return the highest scores (bolded) are shown. Feature groups: SCDM—social support, community, and demographics; SUB—substance use behaviors; TREX—traumatic experiences.

Combinations	Marijuana		Meth		Amphetamines		Cocaine	
Classifiers	LG	DT	LG	DT	LG	DT	LG	DT
Non-injection Drug Use	**0.770** ± 0.010	0.691 ± 0.012	**0.766** ± 0.010	**0.721** ± 0.009	**0.725** ± 0.007	0.642 ± 0.007	**0.759** ± 0.008	**0.776** ± 0.006
	**0.927** ± 0.004	0.883 ± 0.004	**0.910** ± 0.005	**0.872** ± 0.003	**0.679** ± 0.008	**0.543** ± 0.006	**0.715** ± 0.009	0.650 ± 0.007
SCDM+SUB	0.636 ± 0.010	0.719 ± 0.010	0.718 ± 0.010	0.717 ± 0.008	0.655 ± 0.007	**0.648** ± 0.006	0.700 ± 0.007	0.774 ± 0.006
	0.890 ± 0.004	0.894 ± 0.004	0.878 ± 0.005	0.871 ± 0.003	0.622 ± 0.008	0.540 ± 0.005	0.655 ± 0.009	0.650 ± 0.007
SUB+TREX	0.678 ± 0.009	**0.726** ± 0.009	0.752 ± 0.008	0.716 ± 0.008	0.661 ± 0.008	0.646 ± 0.006	0.696 ± 0.008	0.774 ± 0.006
	0.911 ± 0.003	**0.896** ± 0.003	0.904 ± 0.004	0.869 ± 0.003	0.624 ± 0.008	0.539 ± 0.006	0.647 ± 0.009	0.650 ± 0.006
SCDM+SUB+TREX	0.676 ± 0.009	0.715 ± 0.010	0.735 ± 0.008	0.712 ± 0.010	0.657 ± 0.008	0.645 ± 0.006	0.657 ± 0.008	0.775 ± 0.006
	0.910 ± 0.003	0.894 ± 0.004	0.893 ± 0.004	0.869 ± 0.003	0.616 ± 0.008	0.539 ± 0.005	0.600 ± 0.009	**0.651** ± 0.007

Classifier-wise, LG generally performs better than DT across all drugs and all feature selection methods. However, the model with the highest scores for meth prediction was trained under DT. We present further observations and in-depth interpretations of our results shortly in the Discussion section.

#### Predicting increase in drug usage in the next 12 months

Similar to the first predictive problem, we first train a baseline model for each drug using the feature associated with the current usage alone as input. Table 6 shows the results for all considered drugs, classifiers, and feature selection methods. Compared to the performance of the models from the previous problem, the models for predicting an increase in usage seem to perform not as well, with AUROC scores no higher than 0.8 and much fewer models achieving AUROC higher than 0.7. Carefully searching for the optimal (small) subset of features via the top-*k* correlated feature selection method, despite being computationally expensive, helps build models with the highest scores while incorporating very few features (6 or less). The improvements with respect to the baseline models (best among LG and DT) are AUROC by 4.94%, 4.34%, 18.0%, 16.9%, and AUPR by 26.9%, 31.2%, 87.6%, 86.1% for predicting an increase in usage of marijuana, meth, amphetamines, and cocaine, respectively. Again, LG seems to build models with better performance than those from DT in general (Table 6). We provide potential explanations for our observations as well as model interpretations in the Discussion.

**Table 6:** AUROC (top of cell) and AUPR (bottom of cell) measures, averaged over 100 train-test splits, of logistic regression (LG) and decision tree (DT) models for predicting whether PWUDs would exhibit an increase in usage of a certain drug within 12 months using different feature selection methods. The highest scores are bolded.

Methods	Marijuana		Meth		Amphetamines		Cocaine	
Classifiers	LG	DT	LG	DT	LG	DT	LG	DT
Baseline	0.500		0.500		0.500		0.500	
	0.311		0.286		0.202		0.171	
Current Usage as Lone Predictor	0.729 ± 0.008	0.694 ± 0.008	0.714 ± 0.007	0.676 ± 0.006	0.607 ± 0.008	0.531 ± 0.005	0.553 ± 0.008	0.573 ± 0.005
	0.487 ± 0.009	0.464 ± 0.009	0.429 ± 0.008	0.393 ± 0.005	0.242 ± 0.004	0.202 ± 0.001	0.187 ± 0.003	0.187 ± 0.002
Top *k* Mutual Information	0.683 ± 0.009	0.650 ± 0.009	0.667 ± 0.010	0.630 ± 0.009	0.503 ± 0.011	0.483 ± 0.006	0.516 ± 0.010	0.509 ± 0.009
	0.510 ± 0.011	0.438 ± 0.009	0.431 ± 0.010	0.389 ± 0.007	0.244 ± 0.007	0.196 ± 0.003	0.217 ± 0.007	0.182 ± 0.004
Random Forest	0.600 ± 0.012	0.614 ± 0.011	0.583 ± 0.010	0.615 ± 0.010	0.492 ± 0.010	0.488 ± 0.006	0.398 ± 0.011	0.496 ± 0.005
	0.442 ± 0.012	0.413 ± 0.010	0.392 ± 0.009	0.364 ± 0.007	0.233 ± 0.006	0.199 ± 0.003	0.173 ± 0.006	0.173 ± 0.002
Forward Selection	0.511 ± 0.010	0.544 ± 0.011	0.558 ± 0.010	0.510 ± 0.011	0.547 ± 0.010	0.493 ± 0.009	0.484 ± 0.011	0.471 ± 0.011
	0.353 ± 0.008	0.357 ± 0.008	0.371 ± 0.009	0.320 ± 0.007	0.262 ± 0.008	0.226 ± 0.005	0.199 ± 0.005	0.188 ± 0.005
Genetic Algorithm	0.580 ± 0.009	0.548 ± 0.008	0.600 ± 0.009	0.549 ± 0.010	0.497 ± 0.011	0.494 ± 0.010	0.395 ± 0.012	0.496 ± 0.010
	0.408 ± 0.008	0.352 ± 0.006	0.400 ± 0.009	0.344 ± 0.007	0.240 ± 0.007	0.226 ± 0.006	0.174 ± 0.006	0.186 ± 0.004
Manual Grouping	0.709 ± 0.008	0.653 ± 0.008	0.674 ± 0.008	0.666 ± 0.006	0.602 ± 0.009	0.535 ± 0.008	0.566 ± 0.011	0.555 ± 0.007
	0.497 ± 0.011	0.436 ± 0.008	0.436 ± 0.010	0.386 ± 0.004	0.314 ± 0.009	0.229 ± 0.005	0.291 ± 0.009	0.193 ± 0.005
Top-*k* Correlated	**0.765** ± 0.007	**0.720** ± 0.008	**0.740** ± 0.007	**0.745** ± 0.008	**0.716** ± 0.009	**0.628** ± 0.011	**0.670** ± 0.010	**0.593** ± 0.008
	**0.618** ± 0.010	**0.487** ± 0.009	**0.520** ± 0.011	**0.563** ± 0.012	**0.454** ± 0.012	**0.289** ± 0.009	**0.348** ± 0.012	**0.240** ± 0.008

## Discussion

### Population-level substance use behaviors

As observed in Fig 3 and Table 2, most PWUDs reported using multiple drugs, the majority of which include both marijuana and meth. Beside these two drugs, amphetamines and cocaine are also prevalent in our rural population, whereas most injection drugs are not (Fig 2). In terms of substance use dynamics, we observe power-law distributions in both the extent of changes (increase and decrease) for various drugs (Fig 5) and the number of drugs with usage changes (Fig 6). Therefore, the qualitative changes in substance use behaviors within a 12-month period can be characterized by small changes in usage and in the number of drugs used from PWUDs. These findings provide, as far as we are aware, fresh insights into the patterns of short-term changes in substance use behaviors from rural PWUDs at the population level.

### Prediction of individual-level short-term substance use behaviors

#### Predictive question (i)

We now attempt to interpret the LG and DT models for predicting how likely PWUDs would use meth within the next 12 months. We display features for meth in particular because, from the above results in Table 4, the difference in performance between the two classifiers is negligible, and because it is the most commonly used illicit drug according to what we previously observed. Please refer to S4–S9 Tables and S2–S7 Figs for the model interpretation for other drugs. With that, for each classifier, we select the model with features selected from the top-*k* correlated method that returned the highest scores (the same model yields the highest AUROC *and* AUPR for both classifiers of meth) in Table 4 and re-train them using all of the available data in order to update the set of optimal hyperparameters. We then respectively show the significant features along with their corresponding coefficients/weights and plot the learned decision tree of the final LG and DT models trained using all data.

Table 7 shows the features sorted by weight’s magnitude of the final LG model. All features are linked to meth or general drug use, and all are positive factors for using meth within the next 12 months, with currently using meth at high frequency and being incarcerated due to drugs at the top.

**Table 7:** Features from the trained LG model that returns the highest AUROC and AUPR for predicting how likely a PWUD would use meth within the next 12 months.

Weight	Description
+1.29	Meth usage in the past 6 months
+1.06	Being incarcerated due to drugs
+0.82	Generally using meth during evening on an average weekend
+0.63	Generally using meth during afternoon on an average weekday
+0.30	Generally using meth during evening on an average weekday

**Fig 8:**
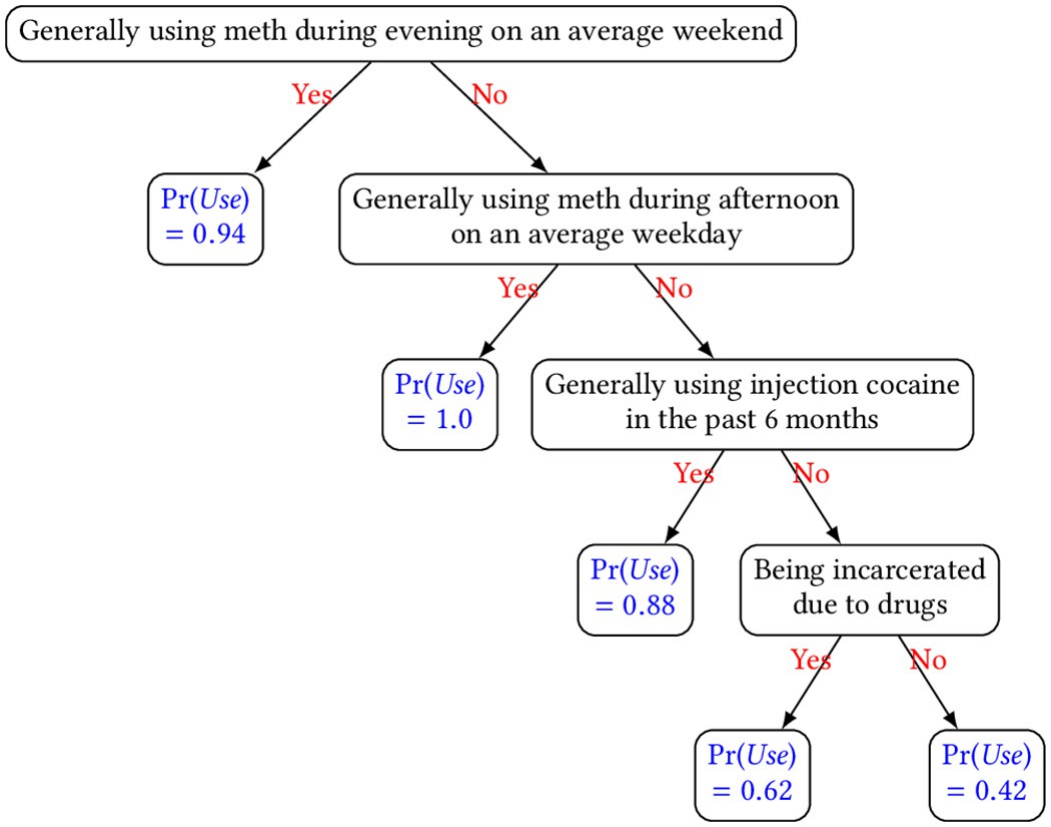
Learned decision tree for predicting how likely a PWUD would use meth within the next 12 months. The probabilities i.e., Pr(*Use*) are displayed at the leaf nodes.

Fig 8 displays the learned decision tree whose structure is consistent with the individual factors from the LG model, which is reasonable as both achieved equally good performance from the above results. Specifically, experiencing drug-related incarceration, using meth during the evening on an average weekend, and using meth during the afternoon on an average weekday all lead to a higher chance of using meth within the next 12 months. One noticeable difference in the tree, nevertheless, is that the current usage of meth is not considered whereas the current usage of injection cocaine is now part of the decision factors, which indicates some form of co-usage of drugs among PWUDs that used meth.

Overall, the significant features from both LG and DT models seem to be sensible as nearly all of them (all for the case of meth) are subject to change over time.

#### Predictive question (ii)

As mentioned earlier regarding our results in Table 6, there is a notable performance drop from the models predicting usage increase compared to their counterparts for question (i). This could be attributed to fewer positive examples during the model training, as can be seen from the drop in performance of the models for amphetamines and cocaine (with only 20% or fewer PWUDs with an increase) compared to those for marijuana and meth (∼30%).

**Fig 9:**
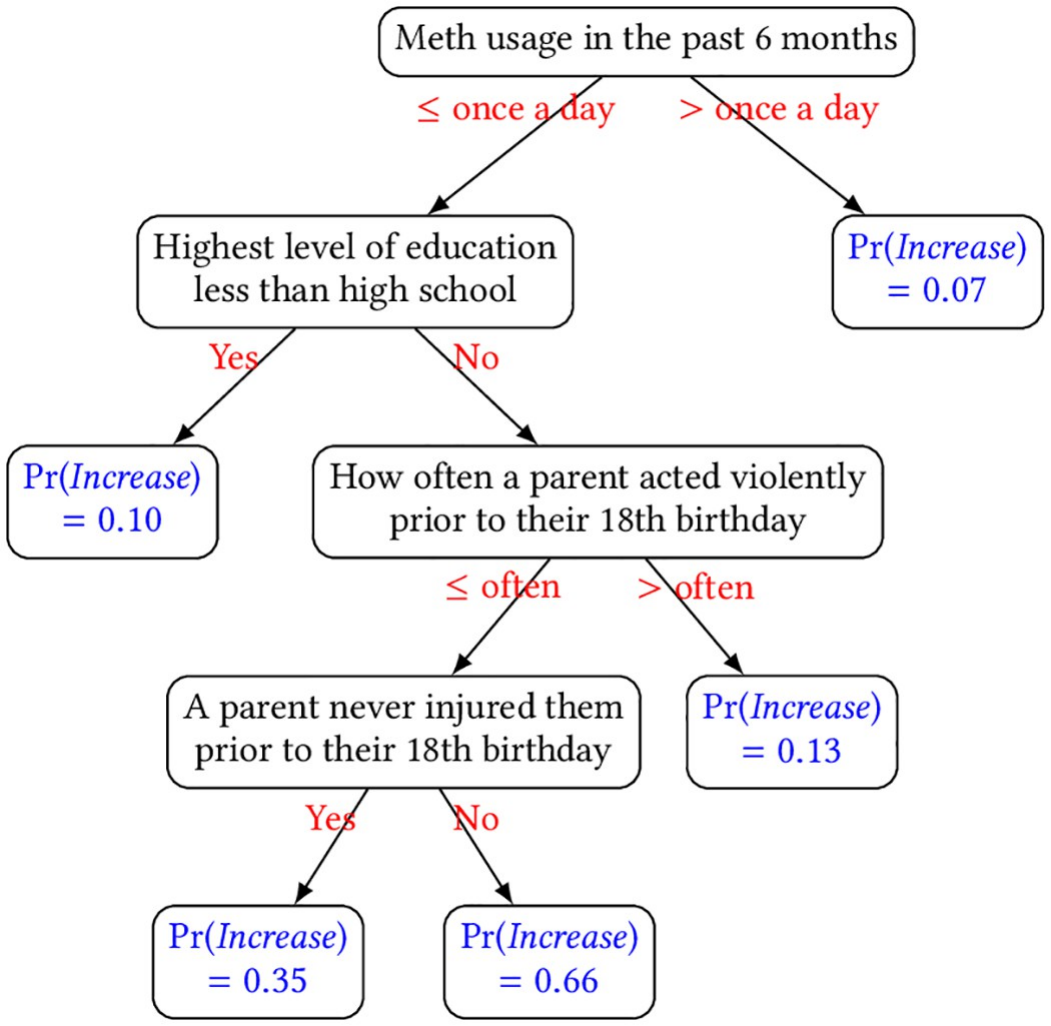
Learned decision tree for predicting how likely a PWUD would increase usage of meth within the next 12 months. The probabilities i.e., Pr(*Increase*) are displayed at the leaf nodes.

To help illustrate how additional features can help improve the performance of baseline models having current usage as their lone predictor, Table 8 and Fig 9 show the features within the trained LG model and the learned decision tree from the DT model for meth, respectively, that returned the highest AUROC (both using top-*k* correlated feature selection method), both of which were trained using all data. We refer readers to S11–S16 Tables for significant features in the models for other drugs. In both models, currently consuming meth at high frequency (more than once a day i.e., either 2–3 times or greater than 4 times a day) reduces the probability of increasing its usage further within the next 12 months, which is reasonable since it would be hard for PUWDs to use more given their already high current usage. Such a relationship could also be attributed to the categorical nature of the usage frequency, for instance, if a PWUD currently uses meth at frequency “4 or more times a day” (the highest possible category in the ordering), they would naturally have a non-increased usage within the next 12 months since there is no category representing frequency higher than that. On another note, we find several unexpected factors/criteria: in LG, cocaine use behaviors have some (negative) impact on the chance of increasing usage of meth; whereas in DT, surprisingly, a low level of education and seeing a parent act violently more than often during childhood lead to low chances of increase.

**Table 8:** Features from the trained LG model for predicting how likely a PWUD would increase meth usage within the next 12 months.

Weight	Description
−0.54	Meth usage in the past 6 months
−0.29	Seen someone overdose on drugs in the past 6 months
−0.19	Generally using cocaine during morning on an average weekend
+0.06	Felt neglected by family prior to their 18th birthday
+0.06	Experienced physical abuse from their mother or female guardian

Overall, although some features of a PWUD’s life are fixed (e.g., adverse childhood experiences) in both LG and DT models, most significant features can change over time and hence can be reliable predictors for an increase in drug use.

#### Increased model complexity does not guarantee performance improvement

In Table 5, although the combination of features on non-injection drug use comprises fewest number of features (as they are also included under SUB group in the other three considered combinations), they constitute models that generally yield the highest predictive performance compared to those built from other combinations. Moreover, the results from Tables 4 and 6 exemplify how models with features selected using conventional feature selection methods may even fail to outperform the baseline models having current usage of a certain drug alone as input. These findings highlight the fact that for our HDLSS setting, increasing a model’s complexity by adding more features does not necessarily improve its generalizability since overfitting is very likely to occur (for which we showed to be the case via learning curves in S1 Appendix and S1 Fig). From the ML literature particularly in medical fields, the idea of so-called “parsimonious” models—those with desired predictive capability from as few inputting features as possible—has been widely embraced and recommended [91–93]. Indeed, for all of our trained models that return the highest scores (all using features selected from the top-*k* correlated method as shown in Tables 4 and 6), the number of considered features ranges from 3 to only 6. Nevertheless, our results as well as the above model interpretations show that additional features, found by exhaustively considering all possible combinations of the top *k* highly correlated features, do help improve the predictive performance of the baseline models having only current usage as a predictor. Despite having high computational cost, we argue that such dedicated and conservative approach is useful to minimize expected overfitting given the HDLSS nature of our data.

### Limitations and caveats

#### Scope of study

Given the employed dataset’s small sample size and heterogeneity (i.e., in which PWUDs were recruited non-randomly via respondent-driven sampling and may or may not have SUDs and other complications in reality), our work on rural PWUDs does not presumptuously aim for specific clinical or intervention applications (e.g., SUD diagnosis), but rather serves as a preliminary study that explores the practicality of ML in providing insightful decision aids toward accurate identification of at-risk individuals. In particular, we aim to identify individual attributes that can complement the baseline models based only on current use of a certain drug for accurately predicting short-term *continuity* as well as *increase* in its usage (via predictive questions (i) and (ii), respectively). The restricted scope of our work toward predictions also means all correlational findings regarding how the features are related to the target variable that we presented during the interpretation of our models are mainly observational and restrictive to populations with similar characteristics and problem definitions under the considered approaches. Therefore, we do not seek to rigorously justify correlation nor causality in the model interpretations, some of which may be contradictory to existing literature or yet to be verified.

#### A data- and problem-driven methodology

Although we opt for a relatively simple treatment of missing data due to negligible differences in predictive performance when we considered more advanced imputation techniques, which could be attributed to data characteristics (i.e., high dimensionality and low missing rate) and the considered data preprocessing step, we still recommend prioritizing more robust methods such as multiple imputation [76, 94] whenever applicable. Furthermore, while the issue of overfitting due to low sample size is reasonably addressed, we stress the importance of rigorous and extensive data manipulations in order to achieve so, and encourage future studies to consider such practices similarly, if not more dedicatedly.

#### Optimality of trained models

Despite achieving promising predictive performance, we do not claim optimality for our trained models in this work since it has been theoretically shown that features that are deemed irrelevant (in terms of their predictive power or correlation with the target variable) by themselves could be useful in conjunction with other features [95]. For a concrete example, the features constructing the DT model that yields highest AUROC for meth include highest level of education, which by itself forms a DT model that returns an abysmal AUROC of 0.534 ± 0.004. When combined with current meth usage, however, the AUROC returned from the resulting DT model substantially improves to 0.702 ± 0.007, which is also higher than the AUROC from the model solely based on current meth usage (0.676 ± 0.006 as shown in Table 6). While exhaustively combining a subset of features allows us to identify useful combinations, the method is computationally expensive given the exponential growth of the power set’s cardinality in terms of the number of considered features *k* (by 2^*k*^). Thus, we expect the existence of unexplored combinations that provide more accurate models than ours.

#### Impact of COVID-19

As stated in the data collection protocol, Wave 1 and Wave 2 of Cohort 1 (consisting of 35 out of 237 PWUDs) were collected prior to and during COVID-19, respectively. The latter wave was administered via a paper booklet that was handed out in a parking lot due to indoor social distancing restrictions. Other than this inconvenience, the more serious COVID-related restrictions in the area had already expired by the fall of 2020, and hence the data collection for Wave 2 remained largely the same as Wave 1. Our quantitative results, however, imply that disturbances caused by the pandemic may have affected the substance use behaviors of PWUDs as we observe slight discrepancies in the prevalence of different drugs (Fig 2) and the number of drugs used by PWUDs (Fig 3) between the two waves.

### Implications

The predictive component of our work can be viewed as a proof of concept through a case study. Given the promising predictive capability of ML for both of our considered problems, we provide a concrete example of practical use cases and some recommendations for effective human-machine collaboration in the context of risk identification and assessment for PWUDs in the following:

1. *Potential application in health care*. Once we obtain a list of drugs a PWUD would most likely use in the short term from the results of predictive question (i) as well as the population-level statistics (e.g., marijuana and meth as the most prevalent drugs), we can further inquire the significant predictors that determine whether they would increase the usage of these drugs via predictive question (ii). We then inform healthcare providers who are seeing clients that are currently using meth and/or cocaine, about the specific predictors to focus on in order to facilitate their identification of at-risk individuals.
2. *Less is more*. Based on our observations and discussion on parsimony when selecting features, even a small set of predictors is sufficient to give satisfactory performance. Therefore, when determining whether a PWUD is at risk from using a certain drug in the short term, we recommend starting with its current usage frequency, then further considering additional predictors suggested by the trained models incrementally.
3. *More is better*. Because substance use likely entails dual-disorders [90] and other complications, we recommend not overlooking seemingly unrelated predictors in the analysis, as they could potentially offer new insights into individual-level substance use behaviors. For instances, in S11 Table, generally using alcohol in the morning on weekdays is associated with decreased chances of increasing marijuana usage within the next 12 months, or in S13 Table, smokeless tobacco use is related to increased chances of escalating cocaine usage in the short term. Given the data-driven approach and the interpretability of our trained models, we believe such comprehensive consideration is practical given sufficient data.

### Future work

Moving forward, we seek to build more informative models, such as predicting future usage frequency using ordinal regression, or ones that incorporate the peer network information from PWUDs (e.g., the extent of drug use among their co-use peers and confidants), which can help further boost decision-making. Regarding the latter, it has been shown that peer networks are also correlated with substance use, e.g., the extent of peer drug use [96], the extent of peer influence [39, 97, 98], the number of substance-using friends [40, 99], the perception of peer usages [100], and peer social norms [101]. Furthermore, there exists the co-evolution of behaviors (i.e., drug use) and peer networks of risk/support [40, 97, 102, 103], in which an individual’s behaviors can change because of peer influence across social network links, and relationship links can break or form because of similarities/differences in the individuals’ attributes (e.g., their drug use behaviors). Thus, it would be interesting to consider models that engage both individual attributes and peer network features in our future work.

## Conclusion

Using longitudinal survey data collected from a sample of 237 drug users in the Great Plains of the U.S., our study provides an in-depth investigation of the short-term changes in substance use behaviors at both the population and individual levels via two respective objectives. For the former, we analyzed our data and accordingly observed that the extent of changes in usage and the number of drugs exhibiting use changes follow power-law distributions, which helped inform healthcare providers, policymakers, and other stakeholders of the extent of the qualitative changes in substance usage of PWUDs over time; for the latter, we defined and addressed two predictive questions of predicting which drug(s) a PWUD would likely use and predicting which drug(s) a PWUD would likely increase their usage within the next 12 months. To tackle each question, we build a multilabel classification model, which consists of separate binary classification models each of which was devoted to a specific drug, particularly marijuana, meth, amphetamines, and cocaine. By combining the resulting models from these two questions, we can create an effective short-term forecasting tool for determining the most appropriate resource (in the form of an intervention program) to allocate to PWUDs that need the most help. Given the interpretability and algorithmic straightforwardness of the individual classifiers, the tool may easily be understood and utilized by human users in health care as a preliminary decision-aid system to improve decision-making toward efficient allocation of limited resources.

## Supporting information

S1 File Survey questions. List of survey questions that form the set of features in Table 1.
(PDF)

S1 Appendix Learning curves. (PDF)

S1 Fig Comparison of learning curves from models that predict whether PWUDs would use meth within the next 12 months using (a, b) small and (c, d) large number of features. Plots (a) and (b) correspond to the LG and DT models with features selected from the top-*k* correlated method that returned the highest scores (the same model yields the highest AUROC and AUPR for both classifiers of meth) in Table 4, which include 5 and 4 features, respectively. Plots (c) and (d) correspond to the LG and DT models that incorporate 165 features from the largest manually-grouped combination (SCDM+ SUB+ TREX). Each plot displays training (red) and cross-validation (green) AUROC as function of number of training examples. The dashed line marks the baseline (all 0.5 for AUROC).
(PDF)

S2 Fig Decision rules from the trained DT model for marijuana. Learned decision tree from the trained DT model that returns the highest AUROC and AUPR for predicting how likely a PWUD would use marijuana within the next 12 months.
(PDF)

S3 Fig Decision rules from the trained DT model for amphetamines. Learned decision tree from the trained DT model that returns the highest AUROC and AUPR for predicting how likely a PWUD would use amphetamines within the next 12 months.
(PDF)

S4 Fig Decision rules from the trained DT model for cocaine. Learned decision tree from the trained DT model that returns the highest (first page) AUROC and (second page) AUPR for predicting how likely a PWUD would use cocaine within the next 12 months.
(PDF)

S5 Fig Decision rules from the trained DT model for opioids. Learned decision tree from the trained DT model that returns the highest AUROC and AUPR for predicting how likely a PWUD would use opioids within the next 12 months.
(PDF)

S6 Fig Decision rules from the trained DT model for injection meth. Learned decision tree from the trained DT model that returns the highest AUROC and AUPR for predicting how likely a PWUD would use injection meth within the next 12 months.
(PDF)

S7 Fig Decision rules from the trained DT model for benzodiazepines. Learned decision tree from the trained DT model that returns the highest AUROC and AUPR for predicting how likely a PWUD would use benzodiazepines within the next 12 months.
(PDF)

S1 Table PWUDs grouped by number of drugs changed in their drug combination. The cumulative percents of PWUDs are with respect to the total number of PWUDs that used at least one drug at either wave (*N* = 230). Within 12 months, more than 80% of PWUDs changed their drug combination by at least one drug.
(PDF)

S2 Table Most prevalent combinations of drugs used by 50% of PWUDs. The respective counts are shown in parentheses and the sum of counts against the total number of PWUDs that used at least one drug is shown in the bottom row.
(PDF)

S3 Table Table 4’s missing results. AUROC (top of cell) and AUPR (bottom of cell) measures, averaged over 100 train-test splits, of LG and DT models for predicting whether PWUDs would use opioids/injection meth/benzodiazepines within the next 12 months using different feature selection methods. The highest scores are bolded.
(PDF)

S4 Table Significant features from the trained LG models for marijuana. Features from the trained LG models that return the highest (left) AUROC and (right) AUPR for predicting how likely a PWUD would use marijuana within the next 12 months.
(PDF)

S5 Table Significant features from the trained LG models for amphetamines. Features from the trained LG models that return the highest (left) AUROC and (right) AUPR for predicting how likely a PWUD would use amphetamines within the next 12 months.
(PDF)

S6 Table Significant features from the trained LG models for cocaine. Features from the trained LG models that return the highest (left) AUROC and (right) AUPR for predicting how likely a PWUD would use cocaine within the next 12 months.
(PDF)

S7 Table Significant features from the trained LG models for opioids. Features from the trained LG models that return the highest (left) AUROC and (right) AUPR for predicting how likely a PWUD would use opioids within the next 12 months.
(PDF)

S8 Table Significant features from the trained LG models for injection meth. Features from the trained LG models that return the highest (left) AUROC and (right) AUPR for predicting how likely a PWUD would use injection meth within the next 12 months.
(PDF)

S9 Table Significant features from the trained LG models for benzodiazepines. Features from the trained LG models that return the highest (left) AUROC and (right) AUPR for predicting how likely a PWUD would use benzodiazepines within the next 12 months.
(PDF)

S10 Table Table 6’s missing results. AUROC (top of cell) and AUPR (bottom of cell) measures, averaged over 100 train-test splits, of LG and DT models for predicting whether PWUDs would exhibit an increase in usage of a certain drug within 12 months using different feature selection methods. The highest scores are bolded.
(PDF)

S11 Table Significant features from the trained LG models for marijuana. Features from the trained LG models that return the highest (left) AUROC and (right) AUPR for predicting how likely a PWUD would increase marijuana usage within the next 12 months.
(PDF)

S12 Table Significant features from the trained LG models for amphetamines. Features from the trained LG models that return the highest (left) AUROC and (right) AUPR for predicting how likely a PWUD would increase amphetamines usage within the next 12 months.
(PDF)

S13 Table Significant features from the trained LG models for cocaine. Features from the trained LG model that returns the highest AUROC and AUPR for predicting how likely a PWUD would increase cocaine usage within the next 12 months.
(PDF)

S14 Table Significant features from the trained LG models for opioids. Features from the trained LG models that return the highest (left) AUROC and (right) AUPR for predicting how likely a PWUD would increase opioids usage within the next 12 months.
(PDF)

S15 Table Significant features from the trained LG models for injection meth. Features from the trained LG models that return the highest (left) AUROC and (right) AUPR for predicting how likely a PWUD would increase injection meth usage within the next 12 months.
(PDF)

S16 Table Significant features from the trained LG models for benzodiazepines. Features from the trained LG models that return the highest (left) AUROC and (right) AUPR for predicting how likely a PWUD would increase benzodiazepines usage within the next 12 months.
(PDF)
