# Supplementary material for: Analyzing and predicting short-term substance use behaviors of persons who use drugs in the great plains of the U.S
Source: PLoS One. 2024 Nov 27;19(11):e0312046. doi: 10.1371/journal.pone.0312046 (PMC11602103; doi:10.1371/journal.pone.0312046)
Supplement: S1 File — List of survey questions that form the set of features in Table 1. (PDF) [file pone.0312046.s001.pdf]

INTRODUCTION - COMMUNITY

These first questions ask about the community you live in.

SC1 How many years have you been living in your current community?

SC3 Overall, how satisfied or dissatisfied are you with living in your current community?

Very dissatisfied
Somewhat dissatisfied
Neutral
Somewhat satisfied
Very satisfied

SC4 Thinking about two years in the future, do you think you will still be living in your current community?

No
Yes

SC5 In the past 6 months, how often have you felt like you belonged in your community? Would you say never, rarely, sometimes, often, or always?

Never
Rarely
Sometimes
Often
Always

SC6 In the past 6 months, how often have you felt out of place in your community? Would you say never, rarely, sometimes, often, or always?

Never
Rarely
Sometimes
Often
Always

SOCIAL SUPPORT

These next questions ask about your social support. For each question, please tell me yes or no if the following statements are true for you.

SC8 There is at least one person that I can confide in.

No
Yes

SC9 There is someone in my life I can get emotional support from.

No
Yes

SC13 There someone who would give me financial assistance.

No
Yes

AGE, GENDER, SEXUAL ORIENTATION DEMOGRAPHICS

Before we go any further, we just need to know a few quick things about you

DM1 How old are you?

TOBACCO

The next questions are about tobacco use.

TB1 Have you ever regularly smoked cigarettes?

- No
- Yes

TB2 How old were you the first time you smoked a cigarette?

- ______ **years old**
- I have never smoked a cigarette -- SKIP TO TB5

TB3 In the past 6 months, have you smoked cigarettes?

- No ⍰ SKIP TO TB5
- Yes

TB4 How many cigarettes do you usually smoke in a day?

- Not at all
- Less than 1 cigarette a day
- 1-5 cigarettes a day
- Half a pack a day
- A pack or more a day

TB5 Have you ever vaped or used electronic cigarettes, also known as e-cigs, with nicotine?

- No ⍰ SKIP TO TB9
- Yes

TB6 How old were you the first time you vaped or used electronic cigarettes, also known as e-cigs, with nicotine?

- ______ **years old**

TB7 In the past 6 months, have you vaped or used electronic cigarettes, also known as e-cigs, with nicotine?

- No ⍰ SKIP TO TB9
- Yes

TB8 In the past 30 days, how many days did you vape or use electronic cigarettes, also known as e-cigs, with nicotine?

- 0 days
- 1-2 days
- 3-9 days
- 10-29 days
- All 30 days

TB9 Have you ever regularly used smokeless tobacco, such as chewing tobacco or snuff?

No SKIP TO NEXT SECTION
Yes

TB10 How old were you the first time you used smokeless tobacco?

______ **years old**

TB11 In the past 6 months, have you used smokeless tobacco?

No SKIP TO NEXT SECTION
Yes

TB12 In the past 30 days, how many days did you use smokeless tobacco?

0 days
1-2 days
3-9 days
10-29 days
All 30 days

ALCOHOL USE

The next questions are about alcohol use. Please remember your answers will be kept private. For these questions, “a drink of alcohol” means a 12 oz beer, a 5 oz glass of wine, or a 1.5 oz shot of liquor

AL1 About how old were you when you first started drinking, including small tastes or sips of alcohol?

[ ]

I have never drank alcohol	88 ▯ SKIP TO NEXT SECTION
Don’t know	99

AL2 About how old were you when you first started drinking, NOT including small tastes or sips of alcohol?

[ ]

Don’t know	99

AL3 About how old were you when you first started drinking to the point of intoxication?

[ ]

Don’t know	99

AL4 In the past 6 months, did you drink any alcohol such as beer, wine, malt liquor, or hard liquor?

No	0
Yes	1

AL5 In the past 30 days, how often did you drink alcohol?

Never → Skip to AL7
Less than once a month
Once a month
2-3 days per month
Once a week
2-3 days per week
4-6 days per week
Everyday

AL6 In the past 30 days, how often did you have 5 or more alcoholic drinks in one sitting?
[4 or more drinks if the respondent is female.]

Never
Less than once a month
Once a month
2-3 days per month
Once a week
2-3 days per week
4-6 days per week
Everyday

INJECTION DRUG USE

The next questions are about injection drug use.

ID1 Have you ever used a needle to inject drugs? This does not include injection of a medical prescription that was being used as intended.

No	0	SKIP TO NEXT SECTION
Yes	1	

ID2 Think back to the very first time you injected any drugs, other than those prescribed for you. How old were you when you first injected any drug?

ID3 In the past 6 months, on average, how often did you inject any drug?

Never □ SKIP TO NEXT SECTION
Less than once a month
Once a month
Once a week
2-6 times per week
One time per day
2-3 times per day
4 or more times per day

ID4 In the past 6 months, how often did you inject heroin?

Never
Less than once a month
Once a month
Once a week
2-6 times per week
One time per day
2-3 times per day
4 or more times per day

ID5 In the past 6 months, how often did you inject prescription opioids?

Never
Less than once a month
Once a month
Once a week
2-6 times per week
One time per day
2-3 times per day
4 or more times per day

ID6 **In the past 6 months, how often did you inject methamphetamine?**

Never
Less than once a month
Once a month
Once a week
2-6 times per week
One time per day
2-3 times per day
4 or more times per day

ID7 **In the past 6 months, how often did you inject cocaine?**

Never
Less than once a month
Once a month
Once a week
2-6 times per week
One time per day
2-3 times per day
4 or more times per day

ID8 **In the past 6 months, how often did you inject a heroin and cocaine speedball?**

Never
Less than once a month
Once a month
Once a week
2-6 times per week
One time per day
2-3 times per day
4 or more times per day

ID9 **In the past 6 months, how often did you inject a heroin and methamphetamine speedball?**

Never
Less than once a month
Once a month
Once a week
2-6 times per week
One time per day
2-3 times per day
4 or more times per day

ID10 In the past 6 months, how often did you inject crack cocaine?

Never
Less than once a month
Once a month
Once a week
2-6 times per week
One time per day
2-3 times per day
4 or more times per day

ID11 In the past 6 months, how often did you inject buprenorphine?

Never
Less than once a month
Once a month
Once a week
2-6 times per week
One time per day
2-3 times per day
4 or more times per day

ID12 In the past 6 months, how often did you inject something else?

Never → Skip to ID14
Less than once a month
Once a month
Once a week
2-6 times per week
One time per day
2-3 times per day
4 or more times per day

ID13 What was the other drug that you injected?

ID14 What kinds of places or locations do you typically inject drugs in, such as a vehicle, at home, or another place?

ID15 In the past 6 months when you injected, how often did you use a new, sterile needle? By a new, sterile needle, I mean a needle never used before by anyone, even you.

Never	0
Rarely	1
About half the time	2
Most of the time	3
Always	4

ID16 In the past 6 months, how often did you use needles that someone else had already injected with?

Never	0
Rarely	1
About half the time	2
Most of the time	3
Always	4

ID17 In the past 6 months have you injected with someone else? By "injecting with someone," we mean you shared drugs, or equipment, or both with at least one other person.

No SKIP TO NEXT SECTION
Yes

ID18 The last time you injected with someone else, did you use a new sterile needle to inject?

No	0
Yes	1

ID19 The last time you injected with someone else, did you use a needle after anyone else had already injected with it?

No	0
Yes	1

ID20 The last time you injected with someone else, was it with one person or with more than one person?

One person	1
More than one person	2 – Skip to ID26

In the past 6 months, how often did you use the following substances? Please DO NOT include any drugs you injected.

ND1 In the past 6 months, how often did you use marijuana?

- Never SKIP TO ND3
- Less than once a month
- Once a month
- Once a week
- 2-6 times per week
- One time per day
- 2-3 times per day
- 4 or more times per day

ND2 How old were you the first time you used marijuana?

ND2A How old were you when you regularly used marijuana?

ND3 In the past 6 months, how often did you use cocaine?

- Never
- Less than once a month
- Once a month
- Once a week
- 2-6 times per week
- One time per day
- 2-3 times per day
- 4 or more times per day

ND4 In the past 6 months, how often did you use Ecstasy or MDMA?

- Never
- Less than once a month
- Once a month
- Once a week
- 2-6 times per week
- One time per day
- 2-3 times per day
- 4 or more times per day

ND5 In the past 6 months, how often did you use PCP?

Never
Less than once a month
Once a month
Once a week
2-6 times per week
One time per day
2-3 times per day
4 or more times per day

ND6 In the past 6 months, how often did you use amphetamines?

Never
Less than once a month
Once a month
Once a week
2-6 times per week
One time per day
2-3 times per day
4 or more times per day

ND7 In the past 6 months, how often did you use methamphetamine?

Never
Less than once a month
Once a month
Once a week
2-6 times per week
One time per day
2-3 times per day
4 or more times per day

ND8 In the past 6 months, how often did you use barbiturates?

Never
Less than once a month
Once a month
Once a week
2-6 times per week
One time per day
2-3 times per day
4 or more times per day

ND9 In the past 6 months, how often did you use benzodiazepines?

Never
Less than once a month
Once a month
Once a week
2-6 times per week
One time per day
2-3 times per day
4 or more times per day

ND10 In the past 6 months, how often did you use opiates/opioids?

Never
Less than once a month
Once a month
Once a week
2-6 times per week
One time per day
2-3 times per day
4 or more times per day

ND11 In the past 6 months, how often did you use heroin?

Never
Less than once a month
Once a month
Once a week
2-6 times per week
One time per day
2-3 times per day
4 or more times per day

ND12 In the past 6 months, how often did you use something else not already listed?

Never
Less than once a month
Once a month
Once a week
2-6 times per week
One time per day
2-3 times per day
4 or more times per day

ND13 What other substance did you use?

ND15 What kinds of places or locations do you typically use non-injection drugs in, such as a vehicle, at home, or another place?

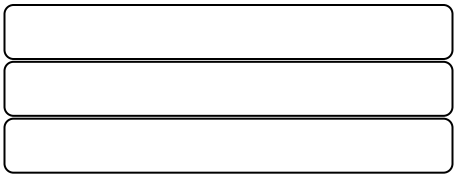

DA1 How easy or hard would you say each of the following drugs are to get?

Marijuana	Very Hard/Hard/Neither Hard nor Easy/Easy/Very Easy
Methamphetamine	Very Hard/Hard/Neither Hard nor Easy/Easy/Very Easy
Cocaine	Very Hard/Hard/Neither Hard nor Easy/Easy/Very Easy
Heroin	Very Hard/Hard/Neither Hard nor Easy/Easy/Very Easy
Opioids/Opiates	Very Hard/Hard/Neither Hard nor Easy/Easy/Very Easy
Benzodiazepines	Very Hard/Hard/Neither Hard nor Easy/Easy/Very Easy
Ecstasy (MDMA)	Very Hard/Hard/Neither Hard nor Easy/Easy/Very Easy

DA2 Do you think there is more or less of each drug available now than there was a year ago?

Marijuana	Much More, More, About the Same, Less, Much Less
Methamphetamine	Much More, More, About the Same, Less, Much Less
Cocaine	Much More, More, About the Same, Less, Much Less
Heroin	Much More, More, About the Same, Less, Much Less
Opioids/Opiates	Much More, More, About the Same, Less, Much Less
Benzodiazepines	Much More, More, About the Same, Less, Much Less
Ecstasy (MDMA)	Much More, More, About the Same, Less, Much Less

DA5 On an average weekday, what time of day do you generally use alcohol? [Check all that apply]

Morning (5AM – Noon)
Afternoon (Noon – 5PM)
Evening (5PM – Midnight)
Night (Midnight – 5AM)
I don’t drink during the week

DA6 On an average weekend, what time of day do you generally use alcohol? [Check all that apply]

Morning (5AM – Noon)
Afternoon (Noon – 5PM)
Evening (5PM – Midnight)
Night (Midnight – 5AM)
I don’t drink on the weekend

DA7 On an average weekday, what time of day do you generally use recreational drugs or drugs not prescribed for you? [Check all that apply]

Morning (5AM – Noon)
Afternoon (Noon – 5PM)
Evening (5PM – Midnight)
Night (Midnight – 5AM)
I don’t use drugs during the week

DA7A On an average weekday, what drug(s) do you generally use during [INSERT TIME]? [Check all that apply]

Marijuana
Cocaine
Ecstasy/MDMA
PCP
Amphetamines
Methamphetamine
Barbiturates
Benzodiazepines
Opiates/Opioids
Heroin
Something else

DA8 On an average weekend, what time of day do you generally use recreational drugs or drugs not prescribed for you? [Check all that apply]

Morning (5AM – Noon)
Afternoon (Noon – 5PM)
Evening (5PM – Midnight)
Night (Midnight – 5AM)
I don’t use drugs on the weekend

DA8A On an average weekend, what drug(s) do you generally use during [INSERT TIME]? [Check all that apply]

Marijuana
Cocaine
Ecstasy/MDMA
PCP
Amphetamines
Methamphetamine
Barbiturates
Benzodiazepines
Opiates/Opioids
Heroin
Something else

DRUG OVERDOSE

The next questions ask about your experiences with drug overdoses.

OD1 Have you ever suffered a drug overdose?

No SKIP TO OD6
Yes

OD2 In the past 6 months, have you ever suffered a drug overdose?

No
Yes

OD6 Do you know anyone who has suffered a drug overdose?

No → skip out of section
Yes

OD7 In the past 6 months, do you know anyone who has suffered a drug overdose?

No
Yes

OD8 Have you ever seen someone overdose on drugs? By seen someone overdose, we mean that you were present when it happened.

No → Skip to OD10
Yes

OD9 In the past 6 months, have you ever seen someone overdose on drugs? By seen someone overdose, we mean that you were present when it happened.

No
Yes

OD10 Do you know anyone who has died from a drug overdose?

No → Skip out of section
Yes

OD11 In the past 6 months, do you know anyone who has died from a drug overdose?

No
Yes

SUBSTANCE USE TREATMENT

Next, I'm going to ask you about alcohol and drug treatment programs. These include outpatient, in-patient/residential, detox, methadone treatment, or 12-step programs.

TX1 Have you been to any of the following drug or alcohol treatment programs? (Y/N)

Outpatient
Inpatient/Residential
Detox
Methadone or Suboxone treatment
12-step alcohol program
12-step drug program
Something else ________

TX2 Are you currently attending or enrolled in any of the following drug or alcohol treatment programs? (Y/N)

Outpatient
Inpatient/Residential
Detox
Methadone or Suboxone treatment
12-step alcohol program
12-step drug program
Something else ________

These next questions ask about things that may have happened in your life before age 18.

AC1A Prior to your 18th birthday, how often did a parent or other adult in the household swear at you, insult you, put you down, or humiliate you?
Never
Once or twice
Sometimes
Often
Very often

AC1B Prior to your 18th birthday, how often did a parent or other adult in the household act in a way that made you afraid that you might be physically hurt?
Never
Once or twice
Sometimes
Often
Very often

AC2A Prior to your 18th birthday, how often did a parent or other adult in the household push, grab, slap, or throw something at you?
Never
Once or twice
Sometimes
Often
Very often

AC2B Prior to your 18th birthday, how often did a parent or other adult in the household hit you so hard that you had marks or were injured?
Never
Once or twice
Sometimes
Often
Very often

AC3A Prior to your 18th birthday, did an adult or person at least 5 years older than you ever touch or fondle you in a sexual way?
No
Yes

AC3B Prior to your 18th birthday, did an adult or person at least 5 years older than you ever have you touch their body in a sexual way?
No
Yes

AC3C **Prior to your 18th birthday, did an adult or person at least 5 years older than you ever attempt to have oral, anal, or vaginal intercourse with you?**
No
Yes

AC3D **Prior to your 18th birthday, did an adult or person at least 5 years older than you ever actually have oral, anal, or vaginal intercourse with you?**
No
Yes

AC4A **Prior to your 18th birthday, how true was the following statement: you felt that no one in your family loved you or thought you were important or special?**
Never true
Rarely true
Sometimes true
Often true
Very often true

AC4B **Prior to your 18th birthday, how true was the following statement: you felt that your family didn't look out for each other, feel close to each other, or support each other?**
Never true
Rarely true
Sometimes true
Often true
Very often true

AC5A **Prior to your 18th birthday, how true was the following statement: you felt that you didn't have enough to eat, had to wear dirty clothes, and had no one to protect you?**
Never true
Rarely true
Sometimes true
Often true
Very often true

AC5B **Prior to your 18th birthday, how true was the following statement: you felt that your parents were too drunk or high to take care of you or take you to the doctor if needed?**
Never true
Rarely true
Sometimes true
Often true
Very often true

AC6 **Prior to your 18th birthday, was a parent/guardian ever lost to you through divorce, abandonment, or other reason?**
No
Yes

AC7A Prior to your 18th birthday, how often was your mother or stepmother or female guardian pushed, grabbed, slapped, or had something thrown at her?
Never
Once or twice
Sometimes
Often
Very often

AC7B Prior to your 18th birthday, how often was your mother or stepmother or female guardian kicked, bitten, hit with a fist, or hit with something hard?
Never
Once or twice
Sometimes
Often
Very often

AC7C Prior to your 18th birthday, was your mother or stepmother or female guardian, ever repeatedly hit over at least a few minutes or threatened with a gun or knife?
No
Yes

AC8A Prior to your 18th birthday, did you live with anyone who was a problem drinker or alcoholic?
No
Yes

AC8B Prior to your 18th birthday, did you live with anyone who used street drugs?
No
Yes

AC9A Prior to your 18th birthday, was a household member depressed or mentally ill?
No
Yes

AC9B Prior to your 18th birthday, did a household member attempt suicide?
No
Yes

AC10 Prior to your 18th birthday, did a household member go to prison?
No
Yes

AC11 Prior to your 18th birthday, how often did other kids, including brothers or sisters threaten you, pick on you or insult you?

Never
Once or twice
Sometimes
Often
Very often

AC12 Prior to your 18th birthday, how often did you feel lonely, rejected, or that nobody liked you?

Never
Once or twice
Sometimes
Often
Very often

AC13 Prior to your 18th birthday, did you live for 2 or more years in a neighborhood that was dangerous, or where you saw people being assaulted?

No
Yes

AC14 Prior to your 18th birthday, was there a period of 2 or more years when your family was very poor or on public assistance?

No
Yes

CRIMINAL JUSTICE INVOLVEMENT

These next questions ask about your experiences with police and incarceration.

CJ1 How concerned are you about the police when using drugs?

- Not at all concerned
- Slightly concerned
- Somewhat concerned
- Moderately concerned
- Extremely concerned

CJ2 How concerned are you about the police when buying or selling drugs?

- Not at all concerned
- Slightly concerned
- Somewhat concerned
- Moderately concerned
- Extremely concerned

CJ3 Have you ever been incarcerated, either jail or prison?

- No → Skip to CJ7
- Yes

CJ4 In the past 6 months, have you been incarcerated?

- No
- Yes

CJ5 How long was your longest incarceration?

- Less than six months
- More than six months but less than a year
- More than a year, but less than two
- From two to five years
- From five to ten years
- More than ten years

CJ6 Have any of your incarcerations been drug related?

- No
- Yes

CJ7 Have you ever received any drug treatment while incarcerated?

- No
- Yes

These next questions are more about you:

DM8 What is your current marital status?

Married
Living together or cohabiting
Separated
Divorced
Widowed
Never married

DM9 What is the highest level of education you completed?

Less than high school
Completed high school or GED
Some college
Completed 2-year degree
Completed 4-year degree
Graduate or professional degree

DM10 What best describes your current employment status? Are you:

Employed full-time
Employed part-time
A homemaker
A full-time student
Retired
Disability - temporary
Disability – permanent
Unemployed
Other (please write in below)

DM12 Do you consider yourself to be Protestant, Catholic, Jewish, Muslim, or something else?

Protestant
Catholic
Jewish
Muslim
None (no religion)
Other,

DM13 [IF DM12 = 0] Within the Protestant faith, do you consider yourself to be:

Evangelical Protestant
Fundamentalist Protestant
Mainline Protestant
Liberal Protestant

Other, [ ]

DM14 How often do you attend religious services?

Never
Less than once a year
About once a year
Several times a year
About once a month
Nearly every week
Once a week
Several times a week

DM15 In general, how much do your religious or spiritual beliefs influence your daily life?

Doesn't apply, not religious or spiritual
Not at all
A little
Some
Quite a bit
Very much

DM17 Have you ever been homeless at any time? By homeless, I mean you were living on the street, in a shelter, in a car, or staying with friends while not paying rent.

No SKIP TO DM20
Yes

DM23 Which of the following categories best describes your total household income in the last 12 months?

Less than $5,000	0
$5,001 to $10,000	1
$10,001 to $20,000	2
$20,001 to $30,000	3
$30,001 to $40,000	4
$40,001 to $50,000	5
$50,001 to $60,000	6
$60,001 to $70,000	7
$70,001 to $80,000	8
$80,001 to $90,000	9
$90,001 to $100,000	10
More than $100,000	

Thank you for completing this part of the survey. Please leave this window open and take the laptop to an interviewer.
