# Supplementary material for: Analyzing and predicting short-term substance use behaviors of persons who use drugs in the great plains of the U.S
Source: PLoS One. 2024 Nov 27;19(11):e0312046. doi: 10.1371/journal.pone.0312046 (PMC11602103; doi:10.1371/journal.pone.0312046)
Supplement: S1 Appendix — (PDF) [file pone.0312046.s002.pdf]

To illustrate how more complex models could potentially cause overfitting, we plotted the learning curves [1], which evaluate the models' generalizability on unseen data subject to the amount of training examples i.e., size of the training set (refer to S1 Fig, which details the chosen models in the caption). In particular, we re-trained each model under the same validation scheme elaborated in our main paper. At each train-test split/iteration, we first find the optimized hyperparameters from the inner 10-fold CV loop, then plot the learning curves as follows:

a. Randomly generate 100 train-test splits, which are distinct from the original splits. These splits are fixed at all iterations.

b. Take 10% of the training set (i.e., 10% of 80% of the entire dataset) and use it to train a model.

c. Compute its score on the training set itself and the held-out test set (remaining 20% of dataset), to which we refer as "training score" and "CV score", respectively.

d. Repeat for all 100 train-test splits then take the average of the scores along with the associated standard deviations (shown as the shaded regions around the means in S1 Fig).

e. Repeat for $20\%, 30\%, \ldots, 100\%$ of training set to get the complete curves.

From the resulting 100 sets of learning curves, we show the one exhibiting the most representative pattern.

In general, when the sample size is small, the training score (shown in red) is high since it is easier for the model to fit on fewer data points. At the same time, the CV score (green) is low as the model encountered insufficient instances and hence could not generalize well to previously unseen data. With more samples being included in the training set, the training and CV scores are expected to gradually converge, which is the case for simpler models consisting of 4-5 features as shown in S1 Fig(a)-(b). If the training scores are high while CV scores are low, and the two scores do not converge, then the model is overfitting the data and in turn generalizes poorly. This behavior can

be clearly observed for complex models that incorporate more than a hundred features as shown in S1 Fig(c)-(d).

References

1. Anzanello MJ, Fogliatto FS. Learning curve models and applications: Literature review and research directions. International Journal of Industrial Ergonomics. 2011;41(5):573–583.
