# Supplementary figures and images for: Analyzing and predicting short-term substance use behaviors of persons who use drugs in the great plains of the U.S
Source: PLoS One. 2024 Nov 27;19(11):e0312046. doi: 10.1371/journal.pone.0312046 (PMC11602103; doi:10.1371/journal.pone.0312046)

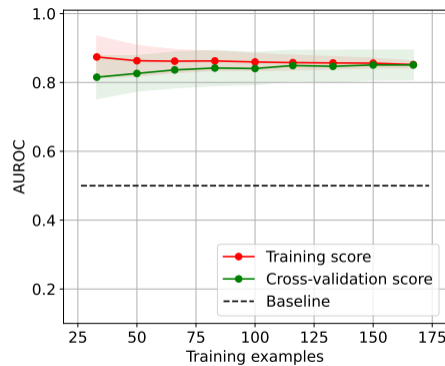

(a)

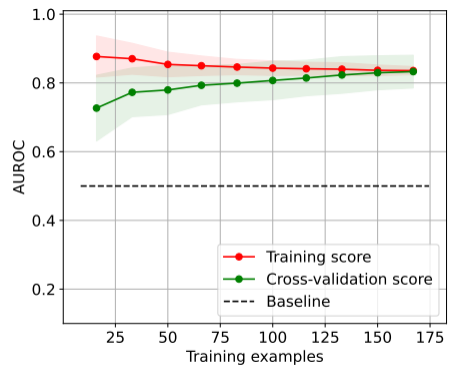

(b)

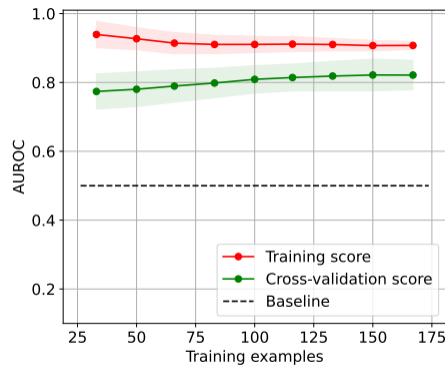

(c)

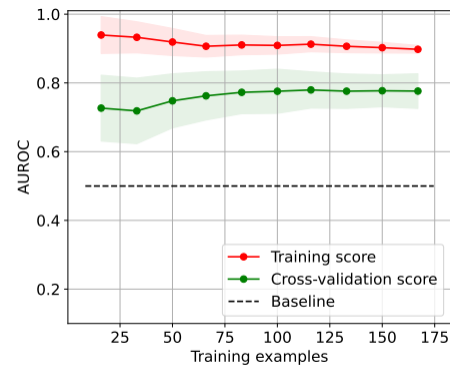

(d)

Supplement: S1 Fig — Plots (a) and (b) correspond to the LG and DT models with features selected from the top-k correlated method that returned the highest scores (the same model yields the highest AUROC and AUPR for both classifiers of meth) in Table 4, which include 5 and 4 features, respectively. Plots (c) and (d) correspond to the LG and DT models that incorporate 165 features from the largest manually-grouped combination (SCDM+ SUB+ TREX). Each plot displays training (red) and cross-validation (green) AUROC as function of number of training examples. The dashed line marks the baseline (all 0.5 for AUROC). (PDF) [file pone.0312046.s003.pdf]

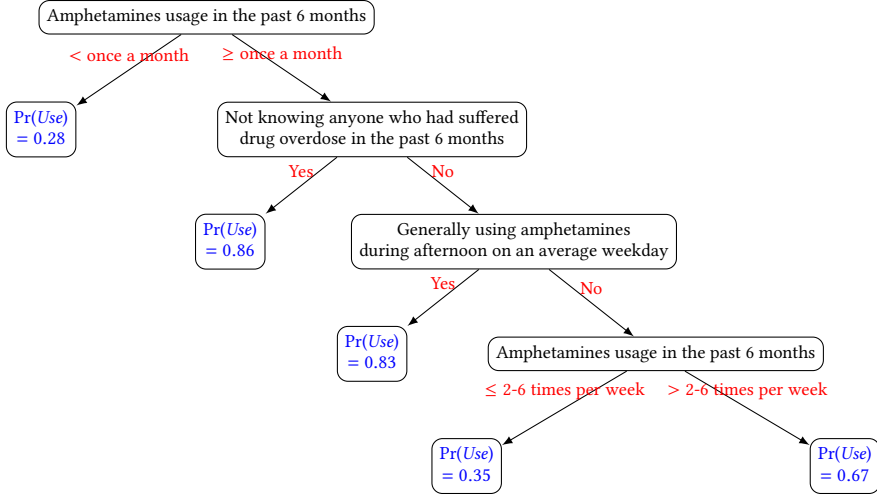

Supplement: S3 Fig — Learned decision tree from the trained DT model that returns the highest AUROC and AUPR for predicting how likely a PWUD would use amphetamines within the next 12 months. (PDF) [file pone.0312046.s005.pdf]

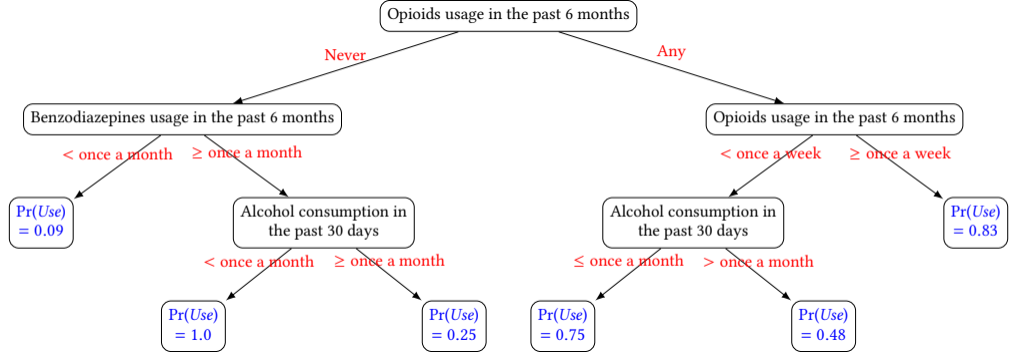

Supplement: S5 Fig — Learned decision tree from the trained DT model that returns the highest AUROC and AUPR for predicting how likely a PWUD would use opioids within the next 12 months. (PDF) [file pone.0312046.s007.pdf]
