# Supplementary material for: Analyzing and predicting short-term substance use behaviors of persons who use drugs in the great plains of the U.S
Source: PLoS One. 2024 Nov 27;19(11):e0312046. doi: 10.1371/journal.pone.0312046 (PMC11602103; doi:10.1371/journal.pone.0312046)
Supplement: S2 Fig — Learned decision tree from the trained DT model that returns the highest AUROC and AUPR for predicting how likely a PWUD would use marijuana within the next 12 months. (PDF) [file pone.0312046.s004.pdf]

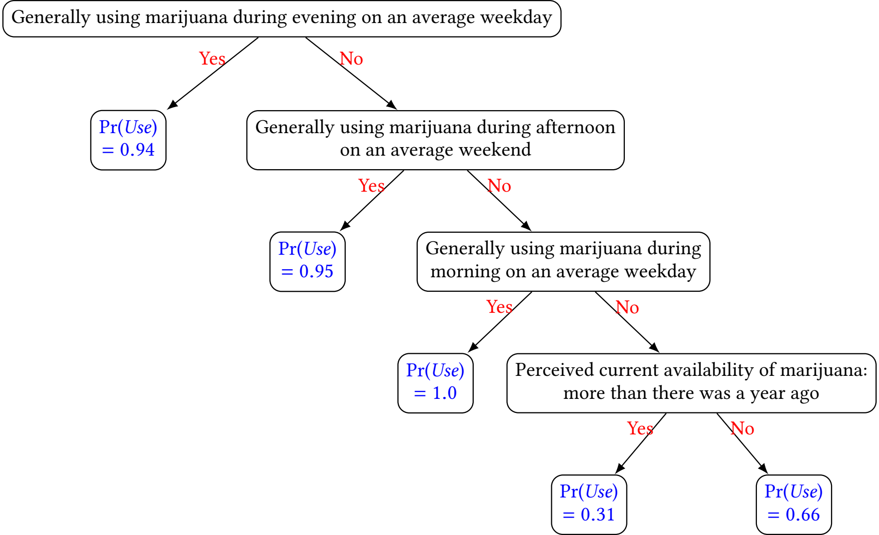
Generally using marijuana during evening on an average weekday
Yes
No
Pr(Use) = 0.94
Generally using marijuana during afternoon on an average weekend
Yes
No
Pr(Use) = 0.95
Generally using marijuana during morning on an average weekday
Yes
No
Pr(Use) = 1.0
Perceived current availability of marijuana: more than there was a year ago
Yes
No
Pr(Use) = 0.31
Pr(Use) = 0.66
