# Supplementary material for: Analyzing and predicting short-term substance use behaviors of persons who use drugs in the great plains of the U.S
Source: PLoS One. 2024 Nov 27;19(11):e0312046. doi: 10.1371/journal.pone.0312046 (PMC11602103; doi:10.1371/journal.pone.0312046)
Supplement: S4 Fig — Learned decision tree from the trained DT model that returns the highest (first page) AUROC and (second page) AUPR for predicting how likely a PWUD would use cocaine within the next 12 months. (PDF) [file pone.0312046.s006.pdf]

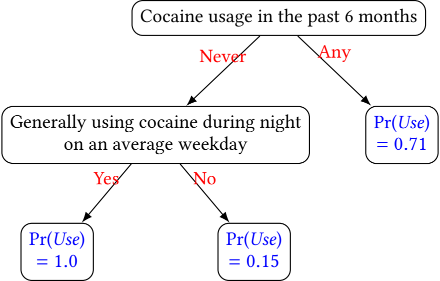
Cocaine usage in the past 6 months
Never
Any
Generally using cocaine during night on an average weekday
Pr(Use) = 0.71
Yes
No
Pr(Use) = 1.0
Pr(Use) = 0.15

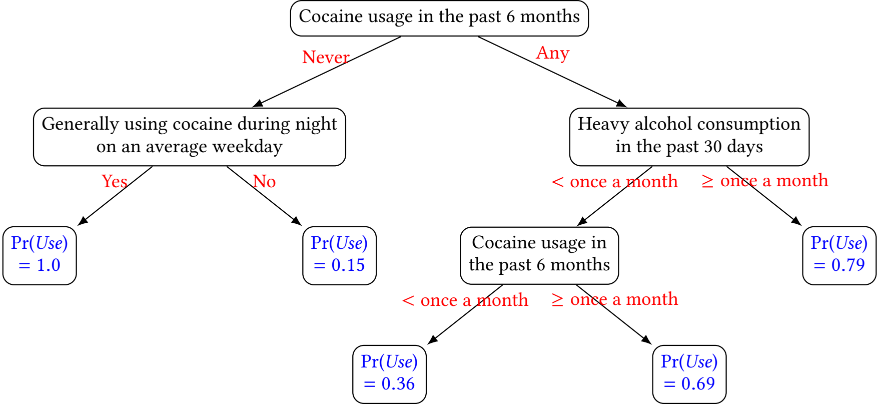

Cocaine usage in the past 6 months
Never
Any
Generally using cocaine during night on an average weekday
Heavy alcohol consumption in the past 30 days
Yes
No
< once a month
≥ once a month
Pr(Use) = 1.0
Pr(Use) = 0.15
Cocaine usage in the past 6 months
Pr(Use) = 0.79
< once a month
≥ once a month
Pr(Use) = 0.36
Pr(Use) = 0.69
