# Supplementary material for: Analyzing and predicting short-term substance use behaviors of persons who use drugs in the great plains of the U.S
Source: PLoS One. 2024 Nov 27;19(11):e0312046. doi: 10.1371/journal.pone.0312046 (PMC11602103; doi:10.1371/journal.pone.0312046)
Supplement: S6 Fig — Learned decision tree from the trained DT model that returns the highest AUROC and AUPR for predicting how likely a PWUD would use injection meth within the next 12 months. (PDF) [file pone.0312046.s008.pdf]

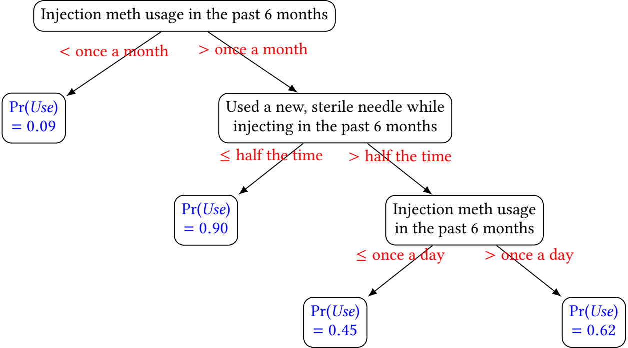

Injection meth usage in the past 6 months
< once a month
> once a month
Pr(*Use*) = 0.09
Used a new, sterile needle while injecting in the past 6 months
≤ half the time
> half the time
Pr(*Use*) = 0.90
Injection meth usage in the past 6 months
≤ once a day
> once a day
Pr(*Use*) = 0.45
Pr(*Use*) = 0.62
