# Supplementary material for: Analyzing and predicting short-term substance use behaviors of persons who use drugs in the great plains of the U.S
Source: PLoS One. 2024 Nov 27;19(11):e0312046. doi: 10.1371/journal.pone.0312046 (PMC11602103; doi:10.1371/journal.pone.0312046)
Supplement: S7 Fig — Learned decision tree from the trained DT model that returns the highest AUROC and AUPR for predicting how likely a PWUD would use benzodiazepines within the next 12 months. (PDF) [file pone.0312046.s009.pdf]

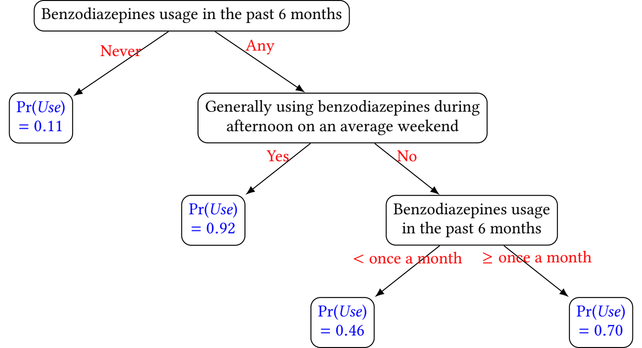
Benzodiazepines usage in the past 6 months
Never
Any
Pr(Use) = 0.11
Generally using benzodiazepines during afternoon on an average weekend
Yes
No
Pr(Use) = 0.92
Benzodiazepines usage in the past 6 months
< once a month
≥ once a month
Pr(Use) = 0.46
Pr(Use) = 0.70
