# Supplementary material for: Analyzing and predicting short-term substance use behaviors of persons who use drugs in the great plains of the U.S
Source: PLoS One. 2024 Nov 27;19(11):e0312046. doi: 10.1371/journal.pone.0312046 (PMC11602103; doi:10.1371/journal.pone.0312046)
Supplement: S1 Table — The cumulative percents of PWUDs are with respect to the total number of PWUDs that used at least one drug at either wave (N = 230). Within 12 months, more than 80% of PWUDs changed their drug combination by at least one drug. (PDF) [file pone.0312046.s010.pdf]

Drug Count	PWUD Count	Cumulative %
1	61	26.5%
2	50	48.3%
3	39	65.2%
4	14	71.3%
5	11	76.1%
6	7	79.1%
7	3	80.4%
8	2	81.3%
9	2	82.2%
10	2	83.0%
>10	2	83.9%
