# Supplementary material for: Analyzing and predicting short-term substance use behaviors of persons who use drugs in the great plains of the U.S
Source: PLoS One. 2024 Nov 27;19(11):e0312046. doi: 10.1371/journal.pone.0312046 (PMC11602103; doi:10.1371/journal.pone.0312046)
Supplement: S2 Table — The respective counts are shown in parentheses and the sum of counts against the total number of PWUDs that used at least one drug is shown in the bottom row. (PDF) [file pone.0312046.s011.pdf]

Wave 1	Wave 2
Marijuana+Meth (20)	Marijuana+Meth (24)
Marijuana+Meth+Inj. meth (16)	Marijuana+Meth+Cocaine (16)
Meth (alone) (11)	Marijuana+Meth+Amphetamines (12)
Marijuana+Meth+Amphetamines+Cocaine (10)	Marijuana+Meth+Inj. meth (11)
Marijuana+Meth+Cocaine (10)	Marijuana+Cocaine (9)
Marijuana+Meth+Amphetamines (7)	Meth (alone) (7)
Marijuana+Meth+Amphetamines+Inj. Meth (6)	Meth+Inj. Meth (7)
Marijuana+Cocaine (5)	Meth+Amphetamines (6)
Marijuana+Opioids (5)	Marijuana+Meth+Cocaine+Inj. Meth (4)
Meth+Amphetamines (4)	Marijuana+Opioids (4)
Marijuana+Meth+Amphetamines+Cocaine+Opioids (4)	Marijuana+Meth+Amphetamines+Opioids (4)
Marijuana+Meth+Amphetamines+Opioids+Benzodiazepines+Barbiturates (3)	Marijuana+Meth+Amphetamines+Cocaine (4)
Marijuana+Meth+Amphetamines+Opioids+Inj. Meth+Inj. opioids (3)	Marijuana+Meth+Amphetamines+Opioids+Benzodiazepines+Inj. Meth (3)
Marijuana+Meth+Amphetamines+Cocaine+Ecstasy (3)	Marijuana+Meth+Amphetamines+Cocaine+Opioids (3)
Meth+Inj. Meth (3)	
Marijuana+Cocaine+Ecstasy (3)	
Marijuana+Meth+Amphetamines+Cocaine+Opioids+Benzodiazepines +Ecstasy+Barbiturates+PCP+Heroin (2)	
115/230	114/227
