# Supplementary material for: Analyzing and predicting short-term substance use behaviors of persons who use drugs in the great plains of the U.S
Source: PLoS One. 2024 Nov 27;19(11):e0312046. doi: 10.1371/journal.pone.0312046 (PMC11602103; doi:10.1371/journal.pone.0312046)
Supplement: S3 Table — AUROC (top of cell) and AUPR (bottom of cell) measures, averaged over 100 train-test splits, of LG and DT models for predicting whether PWUDs would use opioids/injection meth/benzodiazepines within the next 12 months using different feature selection methods. The highest scores are bolded. (PDF) [file pone.0312046.s012.pdf]

Methods	Opioids		Injection Meth		Benzodiazepines	
Classifiers	LG	DT	LG	DT	LG	DT
Baseline	0.500<br>0.321		0.500<br>0.256		0.500<br>0.246	
Current Usage as Lone Predictor	0.797 ± 0.006<br>0.639 ± 0.010	0.773 ± 0.006<br>0.560 ± 0.008	0.846 ± 0.006<br>0.649 ± 0.012	0.818 ± 0.006<br>0.587 ± 0.010	0.805 ± 0.007<br>0.641 ± 0.011	0.789 ± 0.008<br>0.572 ± 0.011
Top k Mutual Information	0.776 ± 0.008<br>0.656 ± 0.010	0.748 ± 0.007<br>0.548 ± 0.009	0.834 ± 0.006<br>0.637 ± 0.012	0.786 ± 0.009<br>0.565 ± 0.011	0.783 ± 0.009<br>0.647 ± 0.011	0.773 ± 0.008<br>0.565 ± 0.011
Random Forest	0.734 ± 0.008<br>0.620 ± 0.010	0.742 ± 0.009<br>0.534 ± 0.010	0.812 ± 0.008<br>0.649 ± 0.012	0.789 ± 0.009<br>0.559 ± 0.012	0.753 ± 0.010<br>0.629 ± 0.013	0.752 ± 0.010<br>0.539 ± 0.013
Forward Selection	0.760 ± 0.008<br>0.644 ± 0.010	0.666 ± 0.011<br>0.506 ± 0.011	0.811 ± 0.007<br>0.623 ± 0.012	0.721 ± 0.010<br>0.516 ± 0.012	0.743 ± 0.010<br>0.597 ± 0.012	0.688 ± 0.011<br>0.505 ± 0.012
Genetic Algorithm	0.702 ± 0.007<br>0.574 ± 0.010	0.659 ± 0.010<br>0.469 ± 0.010	0.794 ± 0.007<br>0.589 ± 0.010	0.673 ± 0.010<br>0.441 ± 0.011	0.724 ± 0.010<br>0.609 ± 0.012	0.653 ± 0.012<br>0.454 ± 0.014
Manual Grouping	0.758 ± 0.007<br>0.664 ± 0.010	0.763 ± 0.006<br>0.552 ± 0.008	0.833 ± 0.006<br>0.674 ± 0.011	0.815 ± 0.007<br>0.585 ± 0.011	0.753 ± 0.008<br>0.639 ± 0.011	0.784 ± 0.007<br>0.563 ± 0.011
Top-k Correlated	$\mathbf{0.817} \pm 0.006$<br>$\mathbf{0.692} \pm 0.009$	$\mathbf{0.779} \pm 0.006$<br>$\mathbf{0.583} \pm 0.010$	$\mathbf{0.850} \pm 0.006$<br>$\mathbf{0.684} \pm 0.011$	$\mathbf{0.819} \pm 0.006$<br>$\mathbf{0.594} \pm 0.011$	$\mathbf{0.823} \pm 0.007$<br>$\mathbf{0.702} \pm 0.011$	$\mathbf{0.791} \pm 0.008$<br>$\mathbf{0.586} \pm 0.011$
