# Supplementary material for: Analyzing and predicting short-term substance use behaviors of persons who use drugs in the great plains of the U.S
Source: PLoS One. 2024 Nov 27;19(11):e0312046. doi: 10.1371/journal.pone.0312046 (PMC11602103; doi:10.1371/journal.pone.0312046)
Supplement: S4 Table — Features from the trained LG models that return the highest (left) AUROC and (right) AUPR for predicting how likely a PWUD would use marijuana within the next 12 months. (PDF) [file pone.0312046.s013.pdf]

Weight	Description
+3.12	Marijuana usage in the past 6 months
+1.44	Being pushed, grabbed, or slapped by a parent prior to their 18th birthday
+1.39	Generally using marijuana during morning on an average weekend

Weight	Description
+2.74	Marijuana usage in the past 6 months
+1.17	Generally using marijuana during evening on an average weekday
+1.12	Being pushed, grabbed, or slapped by a parent prior to their 18th birthday
+0.85	Felt that their family did not look out for, feel close to, or support each other prior to their 18th birthday
