# Supplementary material for: Analyzing and predicting short-term substance use behaviors of persons who use drugs in the great plains of the U.S
Source: PLoS One. 2024 Nov 27;19(11):e0312046. doi: 10.1371/journal.pone.0312046 (PMC11602103; doi:10.1371/journal.pone.0312046)
Supplement: S5 Table — Features from the trained LG models that return the highest (left) AUROC and (right) AUPR for predicting how likely a PWUD would use amphetamines within the next 12 months. (PDF) [file pone.0312046.s014.pdf]

Weight	Description
+1.61	Amphetamines usage in the past 6 months
+0.95	Generally using amphetamines during afternoon on an average weekend
+0.86	Did not know anyone who had suffered drug overdose in the past 6 months
+0.85	Benzodiazepines usage in the past 6 months
−0.76	Perceived current availability of benzodiazepines
+0.56	Been to any 12-step drug treatment program

Weight	Description
+0.72	Amphetamines usage in the past 6 months
+0.49	Did not know anyone who had suffered drug overdose in the past 6 months
+0.41	Been to any 12-step drug treatment program
+0.37	Generally using amphetamines during afternoon on an average weekday
+0.34	Benzodiazepines usage in the past 6 months
−0.04	Being swore at, insulted, or humiliated by a parent prior to their 18th birthday
