# Supplementary material for: Analyzing and predicting short-term substance use behaviors of persons who use drugs in the great plains of the U.S
Source: PLoS One. 2024 Nov 27;19(11):e0312046. doi: 10.1371/journal.pone.0312046 (PMC11602103; doi:10.1371/journal.pone.0312046)
Supplement: S6 Table — Features from the trained LG models that return the highest (left) AUROC and (right) AUPR for predicting how likely a PWUD would use cocaine within the next 12 months. (PDF) [file pone.0312046.s015.pdf]

Weight	Description
+2.41	Cocaine usage in the past 6 months
+1.36	Generally using cocaine during night on an average weekday
+0.88	Heavy alcohol consumption in the past 30 days

Weight	Description
+3.25	Cocaine usage in the past 6 months
+2.67	Ecstasy usage in the past 6 months
+1.45	Generally using cocaine during night on an average weekday
+0.88	Heavy alcohol consumption in the past 30 days
