# Supplementary material for: Analyzing and predicting short-term substance use behaviors of persons who use drugs in the great plains of the U.S
Source: PLoS One. 2024 Nov 27;19(11):e0312046. doi: 10.1371/journal.pone.0312046 (PMC11602103; doi:10.1371/journal.pone.0312046)
Supplement: S7 Table — Features from the trained LG models that return the highest (left) AUROC and (right) AUPR for predicting how likely a PWUD would use opioids within the next 12 months. (PDF) [file pone.0312046.s016.pdf]

Weight	Description
$+3.71$	Opioids usage in the past 6 months
$+1.01$	Felt that they did not have enough to eat, had to wear dirty clothes, and had no one to protect prior to their 18th birthday
-0.17	Alcohol consumption in the past 30 days
$+0.03$	Benzodiazepines usage in the past 6 months

Weight	Description
$+3.51$	Opioids usage in the past 6 months
$+1.00$	Felt that they did not have enough to eat, had to wear dirty clothes, and had no one to protect prior to their 18th birthday
-0.21	Alcohol consumption in the past 30 days
