# Supplementary material for: Analyzing and predicting short-term substance use behaviors of persons who use drugs in the great plains of the U.S
Source: PLoS One. 2024 Nov 27;19(11):e0312046. doi: 10.1371/journal.pone.0312046 (PMC11602103; doi:10.1371/journal.pone.0312046)
Supplement: S8 Table — Features from the trained LG models that return the highest (left) AUROC and (right) AUPR for predicting how likely a PWUD would use injection meth within the next 12 months. (PDF) [file pone.0312046.s017.pdf]

Weight	Description
+2.61	Heroin and cocaine speedball usage in the past 6 months
+2.59	Injection meth usage in the past 6 months

Weight	Description
+2.98	Heroin and cocaine speedball usage in the past 6 months
−2.43	Used a completely new, sterile needle while injecting in the past 6 months
+1.77	Injection meth usage in the past 6 months
