# Supplementary material for: Analyzing and predicting short-term substance use behaviors of persons who use drugs in the great plains of the U.S
Source: PLoS One. 2024 Nov 27;19(11):e0312046. doi: 10.1371/journal.pone.0312046 (PMC11602103; doi:10.1371/journal.pone.0312046)
Supplement: S9 Table — Features from the trained LG models that return the highest (left) AUROC and (right) AUPR for predicting how likely a PWUD would use benzodiazepines within the next 12 months. (PDF) [file pone.0312046.s018.pdf]

Weight	Description
+3.88	Benzodiazepines usage in the past 6 months
+2.12	Generally using benzodiazepines during afternoon on an average weekend
+0.75	Generally using benzodiazepines during afternoon on an average weekday
+0.68	Opioids usage in the past 6 months
−0.09	Generally using benzodiazepines during evening on an average weekday

Weight	Description
+3.23	Benzodiazepines usage in the past 6 months
+1.18	Generally using benzodiazepines during afternoon on an average weekend
+1.04	Generally using benzodiazepines during evening on an average weekend
+0.15	Perceived current accessibility of opioids
