# Supplementary material for: Analyzing and predicting short-term substance use behaviors of persons who use drugs in the great plains of the U.S
Source: PLoS One. 2024 Nov 27;19(11):e0312046. doi: 10.1371/journal.pone.0312046 (PMC11602103; doi:10.1371/journal.pone.0312046)
Supplement: S10 Table — AUROC (top of cell) and AUPR (bottom of cell) measures, averaged over 100 train-test splits, of LG and DT models for predicting whether PWUDs would exhibit an increase in usage of a certain drug within 12 months using different feature selection methods. The highest scores are bolded. (PDF) [file pone.0312046.s019.pdf]

Methods	Opioids		Injection Meth		Benzodiazepines	
Classifiers	LG	DT	LG	DT	LG	DT
Baseline	0.500<br>0.159		0.500<br>0.114		0.500<br>0.129	
Current Usage as Lone Predictor	0.596 ± 0.010<br>0.248 ± 0.008	0.558 ± 0.009<br>0.199 ± 0.004	0.610 ± 0.011<br>0.190 ± 0.006	0.601 ± 0.010<br>0.158 ± 0.004	0.552 ± 0.012<br>0.175 ± 0.007	0.582 ± 0.010<br>0.162 ± 0.005
Top k Mutual Information	0.551 ± 0.011<br>0.248 ± 0.008	0.496 ± 0.008<br>0.179 ± 0.004	0.445 ± 0.012<br>0.150 ± 0.006	0.485 ± 0.009<br>0.125 ± 0.002	0.437 ± 0.013<br>0.166 ± 0.008	0.458 ± 0.008<br>0.120 ± 0.001
Random Forest	0.599 ± 0.009<br>0.292 ± 0.010	0.512 ± 0.005<br>0.181 ± 0.002	0.587 ± 0.011<br>0.213 ± 0.009	0.475 ± 0.003<br>0.119 ± 0.000	0.488 ± 0.012<br>0.172 ± 0.007	0.500 ± 0.005<br>0.126 ± 0.002
Forward Selection	0.635 ± 0.011<br>0.327 ± 0.012	0.530 ± 0.011<br>0.211 ± 0.006	0.446 ± 0.012<br>0.140 ± 0.004	0.495 ± 0.010<br>0.147 ± 0.005	0.395 ± 0.007<br>0.123 ± 0.003	0.493 ± 0.012<br>0.145 ± 0.004
Genetic Algorithm	0.592 ± 0.012<br>0.296 ± 0.012	0.526 ± 0.012<br>0.220 ± 0.009	0.496 ± 0.013<br>0.177 ± 0.008	0.487 ± 0.014<br>0.148 ± 0.006	0.448 ± 0.014<br>0.165 ± 0.008	0.485 ± 0.011<br>0.139 ± 0.005
Manual Grouping	0.684 ± 0.010<br>0.382 ± 0.013	0.582 ± 0.009<br>0.259 ± 0.010	0.622 ± 0.010<br>0.245 ± 0.012	0.565 ± 0.010<br>0.153 ± 0.004	0.602 ± 0.013<br>0.279 ± 0.014	0.566 ± 0.010<br>0.156 ± 0.006
Top-k Correlated	**0.761** $\pm$ 0.009<br>**0.447** $\pm$ 0.015	**0.702** $\pm$ 0.013<br>**0.426** $\pm$ 0.015	**0.761** $\pm$ 0.009<br>**0.401** $\pm$ 0.015	**0.631** $\pm$ 0.009<br>**0.187** $\pm$ 0.011	**0.730** $\pm$ 0.011<br>**0.370** $\pm$ 0.015	**0.647** $\pm$ 0.012<br>**0.266** $\pm$ 0.012
