# Supplementary material for: Analyzing and predicting short-term substance use behaviors of persons who use drugs in the great plains of the U.S
Source: PLoS One. 2024 Nov 27;19(11):e0312046. doi: 10.1371/journal.pone.0312046 (PMC11602103; doi:10.1371/journal.pone.0312046)
Supplement: S11 Table — Features from the trained LG models that return the highest (left) AUROC and (right) AUPR for predicting how likely a PWUD would increase marijuana usage within the next 12 months. (PDF) [file pone.0312046.s020.pdf]

Weight	Description
−1.80	Marijuana usage in the past 6 months
+1.27	Generally using opioids during morning on an average weekend
−1.00	Injected with only one person during the last time injecting with someone else
−0.84	Generally using alcohol during morning on an average weekday
+0.61	Did not suffer drug overdose in the past 6 months

Weight	Description
+2.91	Generally using opioids during morning on an average weekend
−2.18	Marijuana usage in the past 6 months
−1.12	Injected with only one person during the last time injecting with someone else
+1.06	Age started drinking alcohol (not including sips)
−1.05	Generally using alcohol during morning on an average weekday
+0.73	Satisfaction while living in their current community
