# Supplementary material for: Analyzing and predicting short-term substance use behaviors of persons who use drugs in the great plains of the U.S
Source: PLoS One. 2024 Nov 27;19(11):e0312046. doi: 10.1371/journal.pone.0312046 (PMC11602103; doi:10.1371/journal.pone.0312046)
Supplement: S12 Table — Features from the trained LG models that return the highest (left) AUROC and (right) AUPR for predicting how likely a PWUD would increase amphetamines usage within the next 12 months. (PDF) [file pone.0312046.s021.pdf]

Weight	Description
+1.30	Age started injecting any drug
+1.07	Barbiturates usage in the past 6 months
+1.01	Generally using barbiturates during afternoon on an average weekend
+0.94	Generally using alcohol during evening on an average weekend
+0.84	Years lived in their current community
−0.62	An adult attempted to have sexual intercourse with them prior to their 18th birthday

Weight	Description
+3.24	Generally using barbiturates during afternoon on an average weekend
+2.63	Age started injecting any drug
+1.30	Years lived in their current community
+1.14	Generally using alcohol during evening on an average weekend
