# Supplementary material for: Analyzing and predicting short-term substance use behaviors of persons who use drugs in the great plains of the U.S
Source: PLoS One. 2024 Nov 27;19(11):e0312046. doi: 10.1371/journal.pone.0312046 (PMC11602103; doi:10.1371/journal.pone.0312046)
Supplement: S13 Table — Features from the trained LG model that returns the highest AUROC and AUPR for predicting how likely a PWUD would increase cocaine usage within the next 12 months. (PDF) [file pone.0312046.s022.pdf]

Weight	Description
$+3.12$	Age started using smokeless tobacco
-0.98	Never used smokeless tobacco in the past 6 months
-0.83	Perceived current accessibility of meth
$+0.04$	Have regularly used smokeless tobacco
