# Supplementary material for: Analyzing and predicting short-term substance use behaviors of persons who use drugs in the great plains of the U.S
Source: PLoS One. 2024 Nov 27;19(11):e0312046. doi: 10.1371/journal.pone.0312046 (PMC11602103; doi:10.1371/journal.pone.0312046)
Supplement: S14 Table — Features from the trained LG models that return the highest (left) AUROC and (right) AUPR for predicting how likely a PWUD would increase opioids usage within the next 12 months. (PDF) [file pone.0312046.s023.pdf]

Weight	Description
+2.21	Been to any methadone/Suboxone treatment program
−1.64	Perceived current accessibility of heroin
+1.42	Current employment status: permanent disability
+1.27	A household member was depressed or mentally ill prior to their 18th birthday
+0.89	Being threatened, picked on or insulted by other kids prior to their 18th birthday

Weight	Description
+1.37	Been to any methadone/Suboxone treatment program
+1.17	Current employment status: permanent disability
+0.98	A household member was depressed or mentally ill prior to their 18th birthday
−0.80	Perceived current accessibility of heroin
+0.67	Being threatened, picked on or insulted by other kids prior to their 18th birthday
+0.61	Opioids usage in the past 6 months
