# Supplementary material for: Analyzing and predicting short-term substance use behaviors of persons who use drugs in the great plains of the U.S
Source: PLoS One. 2024 Nov 27;19(11):e0312046. doi: 10.1371/journal.pone.0312046 (PMC11602103; doi:10.1371/journal.pone.0312046)
Supplement: S15 Table — Features from the trained LG models that return the highest (left) AUROC and (right) AUPR for predicting how likely a PWUD would increase injection meth usage within the next 12 months. (PDF) [file pone.0312046.s024.pdf]

Weight	Description
−4.51	Generally using cocaine during evening on an average weekend
−2.17	Perceived current accessibility of meth
+1.48	Heroin and cocaine speedball usage in the past 6 months
−1.44	Generally using alcohol during night on an average weekend
−1.09	Have never seen someone overdose on drugs before

Weight	Description
−4.60	Generally using cocaine during evening on an average weekend
−1.87	Perceived current accessibility of meth
−1.50	Generally using alcohol during night on an average weekend
−1.10	Have never seen someone overdose on drugs before
+1.08	Did use a new sterile needle during the last time injecting with someone
+0.95	Felt that their parents were too drunk or high to take care of them prior to their 18th birthday
