# Supplementary material for: Analyzing and predicting short-term substance use behaviors of persons who use drugs in the great plains of the U.S
Source: PLoS One. 2024 Nov 27;19(11):e0312046. doi: 10.1371/journal.pone.0312046 (PMC11602103; doi:10.1371/journal.pone.0312046)
Supplement: S16 Table — Features from the trained LG models that return the highest (left) AUROC and (right) AUPR for predicting how likely a PWUD would increase benzodiazepines usage within the next 12 months. (PDF) [file pone.0312046.s025.pdf]

Weight	Description
+6.01	Current employment status: homemaking
+2.58	Injected with more than one person during the last time injecting with someone else
+1.31	Been to any detox treatment program
+1.00	Generally using amphetamines during afternoon on an average weekend
+0.92	Generally using benzodiazepines during afternoon on an average weekend
+0.91	Opioids usage in the past 6 months

Weight	Description
+3.82	Current employment status: homemaking
+2.30	Injected with more than one person during the last time injecting with someone else
−1.85	Benzodiazepines usage in the past 6 months
+1.44	Generally using benzodiazepines during afternoon on an average weekend
+1.31	Been to any detox treatment program
+1.30	Opioids usage in the past 6 months
+1.11	Generally using amphetamines during afternoon on an average weekend
